# Intermittent Intravaginal Antibiotic Treatment of Bacterial Vaginosis in HIV-Uninfected and -Infected Women: A Randomized Clinical Trial

**DOI:** 10.1371/journal.pctr.0020010

**Published:** 2007-02-23

**Authors:** Taha E Taha, Newton I Kumwenda, George Kafulafula, Bonus Makanani, Chiwawa Nkhoma, Shu Chen, Amy Tsui, Donald R Hoover

**Affiliations:** 1 Departments of Epidemiology and Population, Family and Reproductive Health, Bloomberg School of Public Health, Johns Hopkins University, Baltimore, Maryland, United States of America; 2 Department of Obstetrics and Gynecology, College of Medicine, University of Malawi, Blantyre, Malawi; 3 College of Medicine-Johns Hopkins University Research Project, Blantyre, Malawi; 4 Department of Statistics and Institute for Health, Health Care Policy and Aging Research, Rutgers University, Piscataway, New Jersey, United States of America

## Abstract

**Objective:**

Assess efficacy of intermittent intravaginal metronidazole gel treatment in reducing frequency of bacterial vaginosis (BV).

**Design:**

Randomized, double-masked, placebo-controlled phase 3 trial.

**Setting:**

Postnatal and family planning clinics of the Queen Elizabeth Central Hospital and two health centers in Blantyre, Malawi.

**Participants:**

Nonpregnant HIV-uninfected and -infected women.

**Intervention:**

Intravaginal metronidazole treatment and placebo gels provided at baseline and every 3 mo for 1 y.

**Outcome measures:**

Primary: Cross-sectional and longitudinal comparisons of BV frequency at baseline, 1 mo after product dispensation (post-treatment evaluation [PTE]), and every quarterly visit. Secondary: Effect of treatment on BV clearance and recurrence.

**Results:**

Baseline: 842 HIV-uninfected and 844 HIV-infected women were enrolled. The frequency of BV at baseline in treatment and placebo arms, respectively, was 45.9% and 46.8% among HIV-uninfected women, and 60.5% and 56.9% among HIV-infected women. Primary outcomes: At the PTEs the prevalence of BV was consistently lower in treatment than placebo arms irrespective of HIV status. The differences were statistically significant mainly in HIV-uninfected women. Prevalence of BV was also reduced over time in both treatment and placebo arms. In a multivariable analysis that controlled for other covariates, the effect of intravaginal metronidazole treatment gel compared with placebo was not substantial: adjusted relative risk (RR) 0.90, 95% confidence interval (CI) 0.83–0.97 in HIV-uninfected women and adjusted RR 0.95, 95% CI 0.89–1.01 in HIV-infected women. Secondary outcomes: Intravaginal metronidazole treatment gel significantly increased BV clearance (adjusted hazard ratio [HR] 1.34, 95% CI 1.07–1.67 among HIV-uninfected women and adjusted HR 1.29, 95% CI 1.06–1.58 among HIV-infected women) but was not associated with decreased BV recurrence. *Safety:* No serious adverse events were related to use of intravaginal gels.

**Conclusion:**

Intermittent microbicide treatment with intravaginal gels is an innovative approach that can reduce the frequency of vaginal infections such as BV.

## Editorial Commentary

**Background:** Bacterial vaginosis (BV) results from a change in the normal balance of bacteria in the vaginal tract, and is very common. In pregnant women, it is associated with poorer outcomes in pregnancy, and is also linked with HIV transmission (although it is not certain that BV actually increases the chance of getting HIV—just because these two occur together it does not necessarily follow that one causes the other). BV can be treated with metronidazole tablets, although these can cause gut symptoms and should not be taken repeatedly. The researchers wanted to carry out a multiclinic–based trial to find out whether a metronidazole gel applied intermittently to the vagina (for five nights every three months) would reduce the frequency of BV among women in Malawi. HIV-infected and HIV-uninfected women, recruited from postnatal and family planning clinics, were randomized to receive either metronidazole gels, or equivalent placebo gels, every three months and were then followed up for 12 months. The primary outcome for the trial was the proportion of women with BV at each quarterly follow-up visit, and the researchers intended to compare this outcome between treatment arms at each visit and also to look at the overall changes over time among women receiving either metronidazole or placebo, looking separately at HIV-infected and HIV-uninfected women.

**What this trial shows:** In total 1,686 women took part in the trial (842 not infected with HIV, and 844 infected with HIV). The proportion of HIV-uninfected women with BV dropped by around 20% over the course of the trial, both in women using metronidazole and in those using placebo. However, when comparing the proportion of HIV-uninfected women with BV between the two arms of the trial, there did not seem to be a consistent effect: differences were statistically significant at some time points and not others. Among HIV-infected women, there was also a drop over the course of the trial in the proportion of women with BV, irrespective of whether they used metronidazole or placebo. Again, when comparing the rate of BV among HIV-infected women between study arms (metronidazole versus placebo), the researchers did not see a consistent trend; differences were statistically significant at some time points but not others. Overall, when comparing metronidazole and placebo in an analysis that controlled for other factors, the metronidazole gel seemed to show a small effect in reduction of BV among HIV-uninfected women, but no obvious effect among HIV-infected women.

**Strengths and limitations:** Strengths in the design of this trial include the sample size, which was appropriate to detect an important effect of the metronidazole gel (versus placebo) had one existed, and the randomization and blinding procedures, which were designed to minimize the chance that the trialists or women being enrolled could anticipate to which arm of the trial they might be assigned. A key limitation of this study, as the researchers acknowledge, is the absence of a “no treatment” study arm. The frequency of BV dropped over the course of the trial in women using the placebo gel, raising the possibility that the placebo actually has some effect on bacteria in the vagina. However, a trial with a “no treatment” arm would pose its own problems, since trialists and participants would then not be fully blinded as to their treatment status.

**Contribution to the evidence:** This trial adds data on the efficacy of metronidazole gel when used intermittently, and among women in the community who may or may not actually have BV. Previous studies have evaluated treatment with metronidazole among women who already have symptoms or a diagnosis of BV. The findings of this trial rule out a substantial effect of metronidazole gel, as compared to placebo gel, in reducing the frequency of BV in this setting.

## INTRODUCTION

Bacterial vaginosis (BV) is the most common vaginal infection, and its impact on the health of women is substantial [1–4]. The vaginal ecology is dynamic, where a *Lactobacillus*-dominant flora maintains an optimum acidic pH, which suppresses BV-associated bacteria, and an elevated vaginal pH facilitates growth of sexually transmitted organisms [5–8]. The treatment of choice for BV is oral metronidazole. Another treatment option is intravaginal metronidazole gel (0.75%) [4]. Intravaginal therapy has minimal systemic effects and the cure rates are comparable to the oral regimen [9].

We reported, in studies in Malawi, Africa, that disturbances of vaginal flora were common and associated with HIV-1 infection [10,11]. Identifying a simple treatment regimen for these vaginal disturbances is a priority. Ideally, the treatment of choice should be safe, easy to use, and effective over a long period of time. Because earlier studies showed that only 11% of women had a normal vaginal flora [11], a presumptive (mass) treatment approach would be practical. We therefore decided on an approach in which all women were randomized to receive either an intravaginal antibiotic gel or an intravaginal placebo gel. We opted to use intravaginal metronidazole gel instead of oral metronidazole in this study because oral metronidazole can cause gastrointestinal discomfort and is difficult to tolerate on repeated use [4]. Intravaginal metronidazole gel has a substantially lower dose than oral metronidazole. An additional rationale for use of an intravaginal antibiotic was to mimic other vaginal microbicide studies that require daily use of the product, while in this study use of a vaginal product is intermittent.

## METHODS

### Participants

Nonpregnant HIV-uninfected and -infected women were recruited to this study from the postnatal care and family planning clinics of the Queen Elizabeth Central Hospital, a tertiary referral hospital, and two health centers in Blantyre, Malawi. All women were counseled and gave consent prior to enrollment. Inclusion criteria were: ability and willingness to give written consent; nonpregnant; willingness to return for follow-up visits; willingness to use the product as instructed; willingness to provide specimens at each visit to test for pregnancy and sexually transmitted infection (STI); and residing in the study area. Exclusion criteria were: inability to provide informed consent; pregnant; and refusal of any of the enrollment inclusion requirements. Although use of metronidazole during pregnancy is generally safe, adequate data are not available on use of metronidazole gel during pregnancy. Therefore, women who became pregnant during this study had product use discontinued and were followed throughout pregnancy. This clinical trial was approved by the Johns Hopkins Bloomberg School of Public Health Committee on Human Research (Baltimore, Maryland, United States) and the University of Malawi College of Medicine Research and Ethics Committee (Blantyre, Malawi).

### Interventions

**Figure 1:**
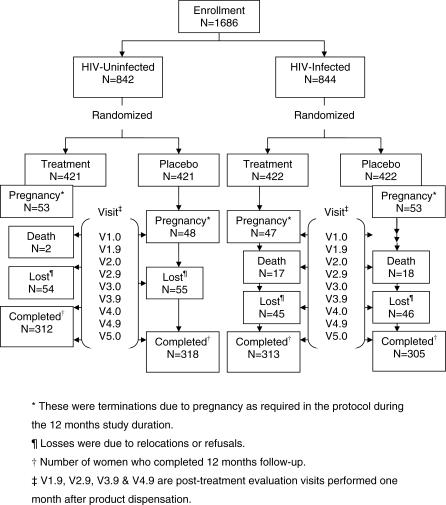
Study Profile

A randomized, double-masked, placebo-controlled, presumptive-treatment, phase 3 clinical trial was conducted. Women were enrolled at the first (baseline) visit (V1.0) and returned for follow-up visits quarterly, at 3, 6, 9, and 12 mo (V2.0, V3.0, V4.0, and V5.0). Additional post-treatment evaluation (PTE) visits occurred 1 mo after product dispensation, at 1, 4, 7, and 10 months (V1.9, V2.9, V3.9, and V4.9) (Figure 1).

### Study Products

We used metronidazole vaginal gel (0.75% MetroGel-Vaginal, 3M Pharmaceuticals, http://www.3M.com/pharma) buffered to a pH of 4.0 in the treatment arm. Each gram of metronidazole vaginal gel contained 7.5 mg of metronidazole and other ingredients as specified by the manufacturer [12]. Each full applicator delivers approximately 5 g of gel containing 37.5 mg of metronidazole in solution. The placebo gel was similar to treatment gel but without the antibiotic metronidazole, and is the same placebo that was used for the licensing studies of the active gel. The placebo gel contained the same excipients as the active gel, including preservatives such as methyl and propyl parapens, as well as edetate disodium. It also contained carbomer 934P, which has a buffering capacity to maintain vaginal pH. Study products were used intravaginally once a day (at bedtime) for five consecutive nights every 3 mo. Participants were counseled in this study not to wash the inside of the vagina until the following morning. At each visit participants received one tube and five disposable vaginal applicators. In total, four courses of product were used during this trial.

### Laboratory Procedures

#### HIV testing.

Prior to enrollment, all women were provided adequate pre- and post-test HIV counseling by trained counselors. HIV testing was performed using either two rapid HIV tests or conventional serologic tests [10]; confirmation with Western blot test was performed for all discordant and new infections.

#### Diagnosis of BV.

The Nugent scoring method (0–3 normal, 4–6 intermediate, and 7–10 BV) [13], based on Gram stained vaginal smears, was used to diagnose BV at each visit. Laboratory examination of the Gram stain slides was performed at the Johns Hopkins University–College of Medicine Research Laboratory in Blantyre, Malawi. For quality control, a random sample of 300 slides was sent for re-evaluation to a reference laboratory in the US (laboratory of S. Hillier, Magee–Women's Research Institute and the University of Pittsburgh, Pittsburgh, Pennsylvania). Agreement between the reference and local laboratory was excellent (kappa 0.89; in the BV classification the two laboratories differed in only five slides; in these, the variability was within two points of the Nugent score).

#### Confirmation of pregnancy.

A commercial kit to detect hCG levels in urine (KAT Quick HCG, KAT Medical, http://www.katmedical.com; the performance of this kit was validated against another commercial kit, QuickVue, Quidel Corporation, http://www.quidel.com) was used to screen for pregnancy at enrollment and each visit. Women with positive urine pregnancy tests were confirmed by ultrasound.

#### Other laboratory tests.

A vaginal wet mount was examined microscopically at each visit to detect motile trichomonads and candida using standard laboratory methods [10]. We did not test for N. gonorrhoeae and *C. trachomatis,* because the prevalence of these organisms has been very low (1%) in recent studies in Malawi [14].

### Study Questionnaires and Physical Examination

Demographic, clinical, acceptability, and adherence questionnaires were completed at enrollment and follow-up visits. To monitor adherence, women were asked to return the empty tube and used applicators at the PTE follow-up visits. The amount of gel used in each tube was also assessed.

### Adverse Experience Reporting

Trained clinicians and nurses conducted a speculum-aided pelvic examination at each visit to detect mucosal abnormalities. Adverse events (AEs) by severity (grade 1, mild; 2, moderate; 3, severe; 4, life threatening; and 5, death) and relatedness to product use were recorded. Serious AEs were defined based on Code of Federal Regulations ICH Guidelines [15].

### Objectives

The primary objective of this study was to determine whether intermittent intravaginal metronidazole gel antibiotic treatment would reduce the frequency of BV among HIV-uninfected and -infected African women. We hypothesized that repeated (intermittent) presumptive treatment with an intravaginal antibiotic regimen would restore normal vaginal flora, enhance BV clearance (conversely, limit persistence), and decrease BV recurrence.

### Outcome Measures

The primary endpoint was proportion of women testing positive for BV based on the Nugent score at each visit, both cross-sectionally (between study arms) and longitudinally (within study arms). The secondary outcome measure was the effect of treatment on clearance and recurrence of BV.

### Sample Size

In this trial a sample size of 832 HIV-uninfected women and 832 HIV-infected women was assumed adequate based on the longitudinal comparisons to provide a power of 87% or more to detect a reduction of 33% or more in the prevalence of BV from a baseline prevalence of 30% (two treatment arms, type 1 error of α = 0.05 and 10% loss during 1 y of follow-up).

### Randomization

Block-randomized computer-generated lists were prepared in the US and were stratified by clinic and HIV status. The study product was provided to the site in a sealed envelope (opaque and padded to avoid damage) with no identifiers other than the study and clinic identification numbers. These envelopes were issued from a central pharmacy at the study site after the women had been counseled and screened, and had given their informed consent to enroll. The study pharmacist and coordinator regularly carried out checks on the order of randomization and matched enrollment identification numbers with clinic and HIV status. The packaging and labeling of the study product was performed in the US by an independent team. Investigators, research workers, and participants were masked about the study product. Neither the study pharmacist at the research site in Malawi or study coordinator was aware of the details of the study product. Both treatment and placebo gels had comparable appearance, consistency, and packaging. Participants were randomized at enrollment (baseline) visit (V1.0).

### Statistical Methods

Data were checked for completeness and consistency and entered locally in a database. All analyses of the primary outcome were performed separately for HIV-uninfected and -infected women using the intent to treat (rather than as treated) approach. The proportion of events occurring in the two study arms were cross-sectionally compared at each visit. Analyses were also conducted to compare events longitudinally within the same study arms between visits. Chi-square (exact test) and other nonparametric tests were used for these comparisons. Generalized estimating equation log binomial models based on relative risk ratio assessed longitudinal associations of treatment with BV after controlling for other covariates measured at multiple visits to account for repeated visit correlation of these repeated observations. Univariable (unadjusted) relative risks (RRs) and adjusted RRs, and 95% confidence intervals (CIs) are presented. Statistical significance was considered to be two-sided *p* ≤ 0.05.

We further analyzed BV clearance and recurrence in the subgroups of women to determine the impact of intravaginal antibiotic treatment among HIV-uninfected and -infected women. We defined BV clearance as a positive BV test at baseline with a negative BV test (first negative test) in a follow-up visit, and BV recurrence as a positive BV test at baseline with a negative BV test (first negative test) in a follow-up visit and becoming BV positive (first positive test) in a subsequent visit. We used Kaplan-Meier survival analyses to estimate median time to clearance and recurrence events and the cumulative probability of these events stratified by study arm among HIV-uninfected and -infected women. Cox proportional hazard models were used to control for various factors and determine the major predictors of these events. Treatment, number of sex partners, frequency of sex, vaginal pH, douching, and T. vaginalis were included in these Cox models (all were time-dependent except for treatment). Statistical significance was determined by a *p*-value ≤ 0.05. All analyses were conducted using SAS (version 8.2; http://www.sas.com).

### Study Monitoring

This study was monitored by a five-member Data and Safety Monitoring Board. Study conduct and review of clinical AEs were also monitored by two independent monitors.

### Provision of Clinical Care

Routine clinical care for all women (including continuous counseling, provision of condoms, and treatment of STIs) was provided by the project at no cost. HIV-infected women were provided additional support by trained counselors and community educators. Specialized clinical care for HIV-infected women was provided by project clinicians and included referral for antiretroviral treatment in a clinic within the Queen Elizabeth Central Hospital in Blantyre.

## RESULTS

### Recruitment

This study was conducted from January 2003 through May 2005. Overall, 3,238 women were screened for this study; of these 1,686 (52.1%) were enrolled: 842 HIV-uninfected and 844 infected women. Of the women screened, 796 (24.6%) were ineligible based on the exclusion criteria and 756 (23.3%) did not consent for HIV testing or did not return to receive their HIV test results.

### Participant Flow

Women who enrolled were equally randomized to each of treatment or placebo arms (Figure 1). Among both HIV-uninfected and -infected women, the proportions discontinued due to pregnancy or lost to follow-up for other reasons occurred comparably between treatment and placebo and were not significantly different (unpublished data). For women who did not complete study visits, all data available prior to discontinuation were included in the analysis.

#### Baseline data.

Baseline sociodemographic and behavioral characteristics were similar in the two study arms both among HIV-uninfected and -infected women (Table 1). There were also no statistically significant differences in baseline characteristics of women who completed the study and those who did not (terminated, died, or lost; unpublished data).

**Table 1:**
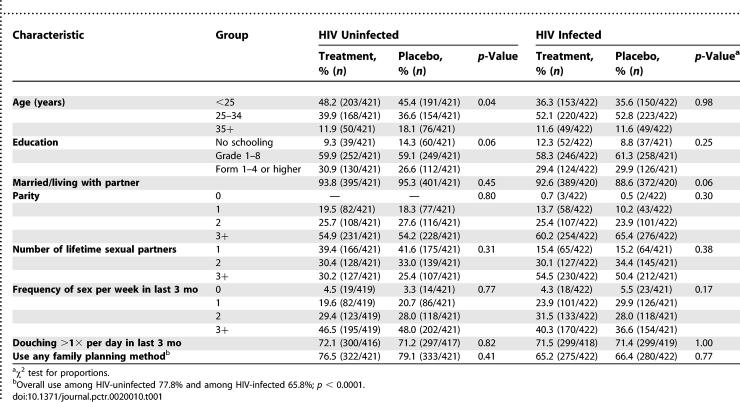
Comparison of Baseline Characteristics among HIV-Uninfected and -Infected Women by Study Arm

### Primary Outcomes

#### Univariable results for HIV-uninfected women.

The visit-specific proportions of women with BV and normal vaginal flora among HIV-uninfected women are shown in Table 2. The prevalence of BV at the enrollment visit (V1.0) was similar in treatment (45.9%) and placebo (46.8%). At the quarterly follow-up visits (V2.0, V3.0, and V4.0), the prevalence of BV was consistently lower in treatment versus placebo; these differences, however, were not statistically significant except at V2.0 (*p* = 0.05). The prevalence of BV dropped over time in both arms compared to baseline to 24.7% in treatment (a drop of 21.2%) and to 28.8% in placebo (a drop of 18.0%) at the last visit (V5.0); *p* < 0.001 (McNemar's discordant pairs). At the PTE visits (V1.9, V2.9, V3.9, and V4.9), the prevalence of BV was consistently lower in treatment than placebo and the differences were statistically significant at each follow-up visit (with the exception of V4.9, *p* = 0.09). The prevalence of normal vaginal flora in treatment was lowest at baseline (V1.0), increased in subsequent visits, and became sustained with a prevalence of over 50% for visits after 6 mo (after V3.0) in quarterly and PTE visits. There were also similar increases in prevalence of normal flora in placebo over time; however, the proportion of women with normal flora was always less in the placebo compared to visit-specific proportions in the treatment arm for both quarterly and PTE visits. The differences in prevalence of normal vaginal flora between treatment and placebo arms were not statistically significant beyond the first initial visits.

**Table 2:**
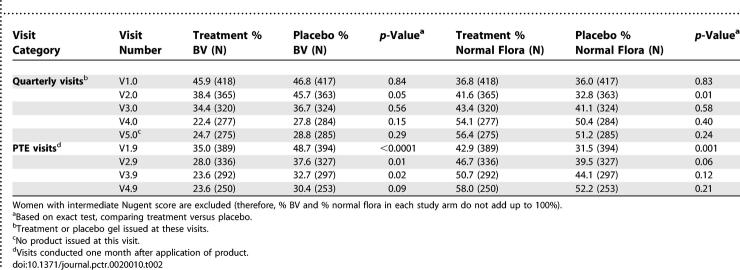
Prevalence of BV and Normal Vaginal Flora by Study Arm among HIV-Uninfected Women

#### Univariable results for HIV-infected women.

Table 3 shows visit-specific proportions of women with BV and normal vaginal flora among HIV-infected women. The prevalence of BV was not statistically different between the two study arms at baseline (although it was higher for HIV-infected than for HIV-uninfected women) and likewise in subsequent quarterly visits. There were significant (*p* < 0.001, McNemar's test) declines in BV prevalence from baseline to V5.0 in both study arms (a drop of 10.9% in treatment and 12.7% in placebo). In follow-up PTE visits, BV was lower in treatment than placebo, but the differences were not statistically significant in the first two PTE visits (V1.9 and V2.9) and became significant in the last two PTE visits (V3.9 and V4.9). There were no statistically significant differences in prevalence of normal flora at the quarterly or PTE visits between treatment and placebo (except V3.9 of PTE). The increase in prevalence of normal flora over time in HIV-infected women was modest compared to the increase in HIV-uninfected women.

**Table 3:**
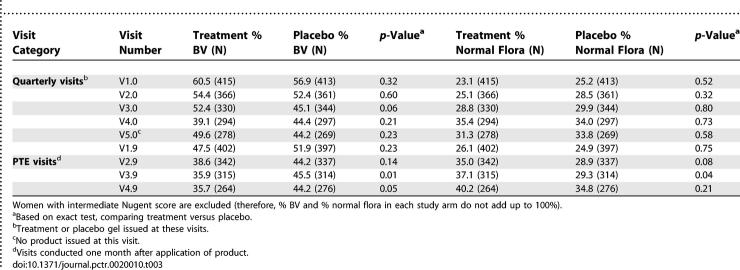
Prevalence of BV and Normal Vaginal Flora by Study Arm among HIV-Infected Women

#### Multivariable results.

The effect of intermittent intravaginal metronidazole gel treatment on BV, compared to the placebo intravaginal gel, was modest in multivariable analyses that controlled for other covariates (Table 4). Among HIV-uninfected women, intravaginal metronidazole treatment significantly reduced the risk of BV after controlling for age, T. vaginalis infection, vaginal pH level, and baseline BV (adjusted RR 0.90; 95% CI 0.83–0.97). Among HIV-infected women, the association of treatment with BV was of borderline significance (adjusted RR 0.95; 95% CI 0.89–1.01). In both HIV-uninfected and -infected women, there were significant associations of BV with the covariates Trichomonas, vaginal pH, and BV at baseline. Detection of Trichomonas and baseline BV was associated with significantly higher risk of BV, while lower vaginal pH was significantly associated with reduced risk of BV (Table 4).

**Table 4:**
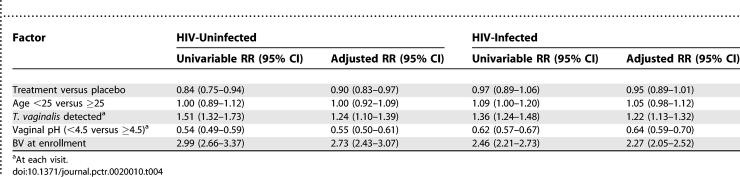
Association of BV with intermittent treatment among HIV-uninfected and infected women

### Secondary Outcomes

#### Clearance of BV.

**Figure 2:**
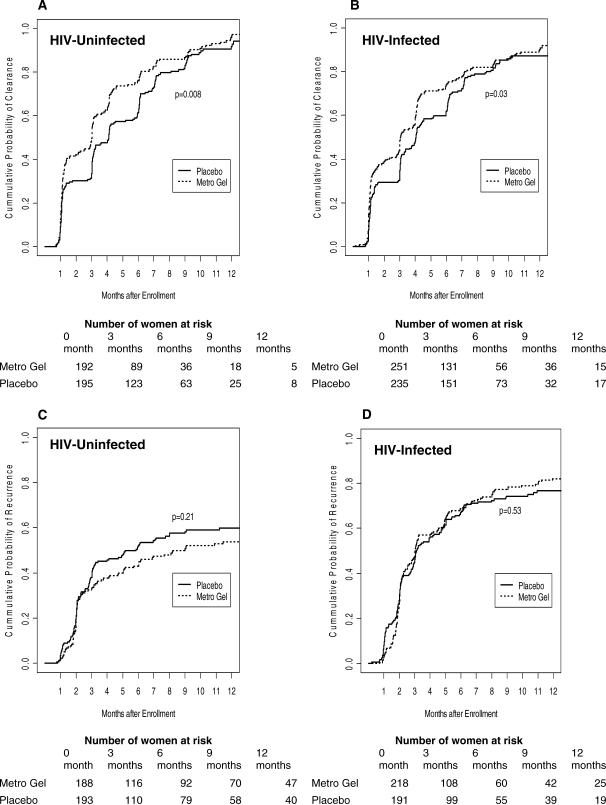
Probability of BV Clearance and Recurrence (A and B) Clearance in HIV-uninfected (A) and -infected (B) women. (C and D) Recurrence in HIV-uninfected (C) and -infected (D) women.

Among 387 HIV-uninfected women who had BV at baseline, 325 cleared BV (165 of 192 in treatment and 160 of 195 in placebo arm). Among 486 HIV-infected women who had BV at baseline 391 cleared BV (209 of 251 in treatment and 182 of 235 in placebo arm). The cumulative probability of BV clearance stratified by study arm in HIV-uninfected and -infected women is shown in Figure 2. In both HIV-uninfected and infected women, the median time to BV clearance among women who had BV at baseline was significantly shorter among those who received metronidazole gel than in those who received placebo gel. For example, among HIV-uninfected women the median of BV clearance in treatment was 92 d compared to 125 d in placebo (*p* = 0.008; log rank test) and among HIV-infected women the median of clearance in treatment was 93 d compared to 123 d in placebo (*p* = 0.03; log rank test). At 3 mo, the cumulative probability of BV clearance among HIV-uninfected women was 49.6% in treatment arm compared to 33.6% in placebo arm, and among HIV-infected women the cumulative probabilities of clearance were similar, 48.6% and 34.8%, respectively. Among both HIV-uninfected and -infected women, treatment with intravaginal metronidazole and lower vaginal pH were significantly associated with increased BV clearance after adjusting for other risk factors (Table 5). On the other hand, Trichomonas infection and multiple sex partners in HIV-uninfected women were significantly associated with decreased BV clearance. Vaginal douching and frequency of sex were not associated with BV clearance after adjusting for other factors.

**Table 5:**
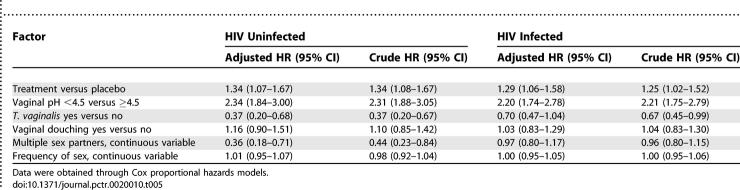
Factors Associated with BV Clearance

#### Recurrence of BV.

We also examined the effect of intravaginal metronidazole gel treatment on BV recurrence among 381 HIV-uninfected and 409 HIV-infected women who had BV at baseline and then cleared BV. Overall, 196 HIV-uninfected women (92 in treatment and 104 in placebo) and 301 HIV-infected women (166 in treatment and 135 in placebo) developed BV recurrence. The cumulative probability of BV recurrence stratified by study arm in HIV-uninfected and infected women is shown in Figure 2. There were no statistically significant differences in median time to BV recurrence between study treatment arms among both HIV-uninfected and -infected women. Among HIV-uninfected women the median time to BV recurrence was 274 d in treatment and 178 d in placebo (*p* = 0.21) and among HIV infected women 92 d in treatment and 95 d in placebo (*p* = 0.53). However, the overall median time to BV recurrence in HIV-uninfected women was significantly longer than in HIV-infected women (210 d versus 93 d; *p* < 0.0001, log-rank test). Among HIV-uninfected women the cumulative probability of BV recurrence at 3 mo was 32.9% in treatment arm and 37.9% in placebo arm. Among HIV-infected women the probability of recurrence at 3 mo was 47.1% in the treatment arm and 46.2% in the placebo arm. Among both HIV-uninfected and -infected women, treatment was not significantly associated with lower BV recurrence. However, low vaginal pH significantly decreased BV recurrence, and vaginal douching significantly increased the risk of BV recurrence, after controlling for treatment and other factors (Table 6). Multiple sex partners and frequency of sex were not associated with recurrence of BV after controlling for other factors.

**Table 6:**
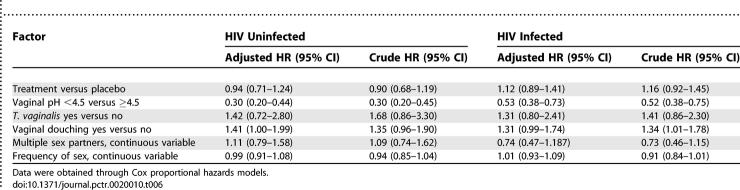
Factors Associated with BV Recurrence

### Adherence and Adverse Events

In this trial, among all women, nonadherence (based on proportion of women who did not return the used tube or used less than 1/3 of the gel in each tube) ranged from 3% to 7% throughout the study and was similar in each study arm. Similarly, acceptability was high (>90%) and was not different in each study arm. There were no statistically significant differences in reported acceptability responses at the last visit prior to loss or discontinuation among women who completed or did not complete the study. There were no serious AEs related to the study product. Among HIV-uninfected women four cases of grade 3 or grade 4 AEs were reported; three in treatment and one in placebo (only one case of symptomatic candidiasis in the treatment arm was considered treatment related). Among HIV-infected women, 28 grade 3 or 4 AEs were reported (17 in the treatment and 11 in the placebo arm; only one case of vulval allergic reaction in the treatment arm was considered related). There were no differences in rates of symptomatic (reported) candida by study arm among HIV-uninfected or -infected women (range 0.5%–5.0%). The frequency of laboratory-confirmed candida infections remained stable in each study arm with minimal fluctuation during study (range 14%–25% in HIV-uninfected women and 18%–29% in infected women).

## DISCUSSION

### Interpretation

The concept of intermittent application of intravaginal products such as a vaginal microbicide is innovative. Nonvaginal intermittent presumptive treatment of STIs has been used in large community-based trials [16]. In this study intermittent treatment with metronidazole gel reduced BV when compared with placebo at the cross-sectional visits or compared longitudinally with baseline frequency. These declines were especially consistent among HIV-uninfected women (Table 2); among HIV-infected women the declines in BV prevalence were less substantial (Table 3). There were also very significant declines from baseline in frequency of BV with placebo, suggesting that the placebo gel may have had a microbicidal effect. It is likely that the placebo, similar to the treatment, may have changed the vaginal environment as a lubricant or chemical barrier. Therefore declines in prevalence of BV with metronidazole gel treatment in this study were in addition to any beneficial effects of placebo.

Among HIV-uninfected women, treatment was associated with a progressive increase in normal vaginal flora (Table 2), suggesting restoration and maintenance of normal vaginal flora. Among HIV-infected women there was no increase in normal flora (Table 3), possibly due to a weaker immune response, inability to restore vaginal flora, or more persistence of BV [17]. In multivariable analysis (after controlling for BV at baseline because it is a predictor of subsequent BV infection), Trichomonas infection increased the risk of BV while lower vaginal pH was associated with lower risk of BV (Table 4). These findings are consistent with vaginal pH patterns in which T. vaginalis and BV organisms favor a higher (>4.5) vaginal pH while other organisms such as candida species favor a lower (4.0–4.5) vaginal pH [7,18]. As reported by others [19,20], the frequency of candida was higher among women who used metronidazole gel, but most of these women were asymptomatic. Treating BV-associated bacteria may also allow other organisms such as candida species to predominate [21].

Examination of BV prevalence pattern in Tables 2 and 3 shows that there was a decrease in BV prevalence following each act of intravaginal gel application (PTE visits) that was followed by an increase at each subsequent quarterly visit (e.g., in Table 2 in the treatment arm BV prevalence increased from 35.0% at V1.9 to 38.4% at V2.0 and from 28.0% at V2.9 to 34.4% at V3.0). This suggests either BV recurrence or persistence (i.e., inadequate clearance of BV). Our findings suggest that metronidazole intravaginal gel, compared to placebo, was more effective at clearing BV than preventing recurrences. For example, after controlling for several factors, treatment with metronidazole gel in both HIV-uninfected and HIV-infected women was significantly associated with BV clearance (Table 5) but not with BV recurrence (Table 6). Among both HIV-uninfected and -infected women the median time to clearance of BV was about three months with metronidazole gel and about four months with placebo gel (*p* < 0.05). The median times for recurrence were not different by study arm (albeit significantly shorter among HIV-infected [∼3 mo] compared to uninfected women [∼7 mo]). Vaginal douching was not associated with BV clearance but significantly increased recurrence. It is likely that BV clearance was not influenced by vaginal douching, because women were advised not to douche after application of the intravaginal gel. Because douching is a common practice in this population (∼70% reported douching at baseline), women would have resumed regular douching after completion of gel use, enhancing BV recurrence, possibly through changes in vaginal flora [22].

The lack of a study arm that did not use an intravaginal gel as a control arm is a limitation of this study, as an estimate of true efficacy of the metronidazole intravaginal treatment gel would have required use of no product. This is a challenging aspect of almost all microbicide trial designs [23]. As shown in Table 4 the overall estimate of efficacy of treatment gel compared to placebo gel was modest. Use of an arm with no intravaginal gel, however, would have weakened the study design due to nonmasking, possible dilution of estimated effect (due to potential contamination), and poor retention. The alternative design of using intermittent oral metronidazole tablets as a comparison arm also has limitations such as nonmasking. Other factors, such as changes in behavior for being in the study, should also be considered for the substantial BV reductions (Hawthorne effect). It is unlikely, however, that the temporal effect of any counseling and clinical services provided during this study has been differential, because these services were for all women and the study personnel were masked. The most plausible explanation for the similar trends in BV reduction in both arms of this study is the inherent activity of the placebo formulation. Despite metronidazole intravaginal gel being more effective, its true efficacy is underestimated in this study due to the residual effect of the placebo gel. Finding a universal “inert” placebo vaginal gel remains a priority for microbicide research.

The results of this study are unlikely to be influenced by losses of women during follow-up. The losses in each study arm were comparable and there were no differences in demographic and acceptability characteristics of women who completed or did not complete the study. Additionally, the efficacy of treatment was established by both cross-sectional and longitudinal analyses when losses were minimal at earlier visits. In this study, we note that a comparable proportion of HIV-infected and -uninfected women became pregnant. Other studies in sub-Saharan Africa, however, reported lower rates of pregnancy among HIV-infected women due to biological factors limiting fertility in HIV-infected women such as failure to conceive and increased pregnancy losses [24]. Further analysis of our data suggests that multiple factors may have contributed to the comparability of pregnancy rates among HIV-infected and -uninfected women. For example, over 70% of the HIV-uninfected women were using a family planning method (Table 1), prevalence of use was significantly higher among HIV-uninfected compared to -infected women (*p* < 0.0001), and additionally, HIV infected women who became pregnant were younger, healthier, wanted a pregnancy sooner, and used a family planning method less (unpublished data). We did not assess drug resistance during this intermittent use of the product. Available data are reassuring and suggest that metronidazole is highly active despite its widespread use and development of resistance is rare [25].

Intermittent use of intravaginal gels should be considered for treatment of vaginal infections. These gels could modify the vaginal milieu, coat vaginal surfaces and potentially prevent attachment of pathogenic organisms to appropriate receptors, and/or physically reduce itching and accompanying trauma inside the vagina or on the vulval surface. Vaginal products, mostly traditional agents, are extensively used in sub-Saharan Africa [26]. Therefore, intermittent use of vaginal gels would be culturally acceptable.

### Generalizability

The findings of this study can be applied to other populations in Africa. Despite the exclusions we made in this trial as required in the protocol, women enrolled had important characteristics comparable to other populations in Malawi and sub-Saharan Africa, thus enhancing external validity. For example, HIV prevalence among women enrolled in this study was around 26% (similar to HIV prevalence estimates in the antenatal/postnatal clinics in urban Malawi [27]) and BV prevalence in this study was similar to others reported from Uganda and South Africa [1,2].

### Overall Evidence

BV is a common condition with a poorly understood etiology [28]. It is not recognized as a typical STI despite being influenced by multiple underlying risk factors that are characteristic of conventional STIs. Likewise, its treatment and prevention remain difficult. We conducted a web-based PubMed (http://www.pubmed.gov) literature search focusing on treatment of BV in nonpregnant women to compare with findings from our current trial. In a review article of BV treatment options in nonpregnant women, Joesoef and colleagues recommended oral metronidazole (500 mg twice daily for 7 d), clindamycin vaginal cream (2% once daily for 7 d), or metronidazole vaginal gel (0.75% twice daily for 5 d) [29]. Subsequent to this review, a randomized prospective study conducted in the US reported no differences in efficacy or safety between once-daily or twice-daily BV treatment for five consecutive days with 0.75% metronidazole intravaginal gel [20]. Comparison of intravaginal and oral metronidazole treatment regimens also showed no differences in efficacy by mode of administration; however, women receiving oral metronidazole are more likely to experience gastrointestinal side effects, although oral medication may be more convenient [29]. We opted to use a once-daily intravaginal regimen for five days to simplify the regimen and maximize adherence. We also preferred a topical application instead of oral medication because of our interest in vaginal microbicides and assessment of the intermittent regimen in an African setting where adherence with a daily application of an effective vaginal microbicide could be a challenge. The findings of this study are in agreement with available literature on the efficacy of treatment of BV using intravaginal metronidazole gel [29]. The longitudinal efficacy estimates of intravaginal metronidazole gel in our study were lower than estimates reported from US studies because we followed a presumptive treatment approach and did not limit enrollment to symptomatic or BV-established women as has been the approach in other studies [29]. Recurrence of BV is a limitation of existing regimens, and recent data suggest that a more aggressive and extended treatment is needed to manage BV recurrences [30]. Our study is unique in assessing the concept of intermittent intravaginal gel and for evaluating this regimen among both HIV-uninfected and HIV-infected African women.

## SUPPORTING INFORMATION

CONSORT Checklist Click here for additional data file. (51 KB DOC)

Trial Protocol Metro Study Protocol 2001 (9.6 MB PDF)

## Abbreviations

BV: bacterial vaginosis
HR: hazard ratio
PTE: post-treatment evaluation
RR: relative risk
STI: sexually transmitted infection
