# Supplementary material for: Intermittent Intravaginal Antibiotic Treatment of Bacterial Vaginosis in HIV-Uninfected and -Infected Women: A Randomized Clinical Trial
Source: PLoS Clin Trials. 2007 Feb 23;2(2):e10. doi: 10.1371/journal.pctr.0020010 (PMC1851729; doi:10.1371/journal.pctr.0020010)
Supplement: Trial Protocol — (9.6 MB PDF) [file pctr.0020010.sd002.pdf]

* Protocol

Taha Taha

INTRAVAGINAL TREATMENT OF DISTURBANCES OF VAGINAL FLORA AMONG HIV INFECTED AND UNINFECTED WOMEN IN MALAWI

Principal Investigators

Taha E. Taha MD PhD, Department of Epidemiology, School of Hygiene and Public Health, Johns Hopkins University; Rm E6140; 410 614 5255; ttaha@jhsph.edu

Newton Kumwenda PhD, Field Director, Johns Hopkins University Research Project, P.O. Box 1131, Blantyre, Malawi; jhopkins@sdnp.org.mw

Co-investigators

George Liomba MBBS FRCPath, College of Medicine, University of Malawi

Donald Hoover PhD, Rutgers University, NJ (Protocol Statistician)

Armelia Chaponda RN MPH, Johns Hopkins University Research Project (Study Coordinator)

Aleen McClure BS MS, Johns Hopkins University Research Project (Study Coordinator)

2001

Copy of protocol approved by CHR

RESEARCH PLAN

A. SPECIFIC AIMS

Vaginal flora disturbances are common among women of reproductive age in both developed and developing countries. In Malawi, only 11% of childbearing women had a normal vaginal flora and 89% had a vaginal disturbance which ranged from mild to severe.[1] Several adverse reproductive outcomes are associated with "bacterial vaginosis (BV)", a severe disturbance of vaginal flora. Therefore, identifying a simple treatment regimen for these vaginal disturbances is highly desirable for Malawi and similar developing countries. We propose a randomized, double-masked, placebo-controlled clinical trial of use of an intravaginal antibiotic (0.75% metronidazole gel) for mass treatment of vaginal disturbances. The specific aims of this study are:

<u>Primary</u>

1. To determine the effect of postnatal intravaginal metronidazole gel treatment (once daily for 5 consecutive days every three months) on the incidence of BV among HIV infected and uninfected women. We hypothesize that in areas where BV is common, an intermittent treatment regimen will restore normal flora, reduce vaginal disturbances and their recurrences. Such a measure is simple and woman-controlled. This intervention has the potential to reduce adverse reproductive outcomes and improve maternal health.

2. To determine the effect of mass treatment of BV on rates of conception and early pregnancy loss among HIV infected and uninfected women of reproductive age. An association between BV and miscarriage has been reported and HIV is known to be associated with pregnancy loss. Therefore, among populations where the prevalences of BV and HIV are high, such as in sub-Saharan Africa, the assessment of the independent effect of these conditions on conception and pregnancy loss is important. We hypothesize that treatment of BV will reduce early pregnancy loss among both HIV infected and uninfected women.

<u>Secondary</u>

3. To assess the clinical and behavioral factors which facilitate or impede the long-term use of metronidazole vaginal gel in a community where use of traditional vaginal agents is common. This product has minimal systemic or local side effects compared to oral metronidazole. We will obtain qualitative and quantitative behavioral data to evaluate acceptability and compliance. Study of these factors is critical to evaluate the overall efficacy of the intervention.

---

[1] Disturbances of vaginal flora were defined as mild when only one of four clinical criteria (vaginal pH>4.5, increased homogenous vaginal discharge, positive amine test and clue cells≥20%) was present; moderate when two criteria were present, and severe when three or more criteria were present. In this proposal, we follow these definitions and use the terms severe disturbance and bacterial vaginosis (BV) interchangeably.

B. BACKGROUND AND SIGNIFICANCE

BACKGROUND

1. Bacterial Vaginosis: Epidemiology and pathogenesis

Bacterial vaginosis (BV) is the commonest vaginal infection among women of reproductive age in the United States (1). Among African women, prevalence rates of more than 50% have been reported (2,3). It is a syndrome characterized by changes in the vaginal flora resulting in loss of lactobacilli, an increase in other predominantly anaerobic flora and an increase in vaginal pH (4). In the healthy vagina, lactobacilli predominate and form about 95% of the total bacterial count. Hydrogen peroxide (H_2O_2) producing strains of lactobacilli are found in about 96% of women with normal vaginal microflora, but only in about 6% of women with BV, suggesting an important protective role against BV by the (H_2O_2) producing lactobacilli (5). Hydrogen peroxide alone can inhibit growth of bacteria lacking the enzyme catalase, and in conjunction with other host enzymes such as peroxidase present in the cervix and endometrium, can produce a potent bactericidal mechanism (6). The acidic environment of the normal vaginal flora among women of reproductive age is attributed to lactobacilli which produce lactic acid from glucose as a result of glycogen metabolism. This process keeps the normal vaginal pH at a range of 3.2-4.2, and in turn, maintains the existence of a lactobacillus-dominant normal microflora. The acidic environment inhibits colonization by potentially pathogenic bacteria while an elevated pH facilitates growth of sexually transmitted disease (STD) agents such as *T. vaginalis* (7-9). In addition to bacterial infections, women with higher vaginal pH such as in BV could be either more susceptible to HIV infection, or more infectious to their partners due to increased titer of infectious virus (10,11). In summary, the vaginal ecology is dynamic; a *lactobacillus*-dominant flora maintains an optimum acidic pH which suppresses BV associated bacteria (e.g., G. *vaginalis*, *Bacteroides* spp., *Mobiluncus* spp., M. *hominis* and *Peptostreptoccus*).

BV, however, represents the severest form of vaginal disturbance and intermediate stages of vaginal flora have been described. Intermediate flora may represent a transitional pattern. Women with intermediate flora are more likely to advance to BV than revert to a normal flora state (4). Among 171 pregnant women in labor at term, H_2O_2 producing lactobacilli were recovered from 5% of women with BV, from 37% with intermediate flora and from 61% with normal flora (12). Different levels of STDs and other vaginal infections were also observed to vary among women with different patterns of vaginal flora. For example, Group B *streptococcus* and yeast were more frequently recovered from normal or intermediate flora, *T. vaginalis* was more common in intermediate flora, and N. *gonorrhoeae* and C. *trachomatis* were associated with intermediate or severe vaginal disturbance (8). The role of sexual transmission of BV has not been conclusive. In several studies consistent associations of BV with number of sexual partners and various forms of sexual practices have been shown (1,13). However, treatment of male partners did not prevent recurrence of BV (14,15), suggesting that BV is unlikely to be sexually transmitted. Despite being described as an infection, BV does not produce an inflammation and polymorphonuclear leucocytes are not present in large amounts (1) as is the case with STDs.

2. Association of bacterial vaginosis with adverse reproductive outcomes

BV has been implicated in several serious obstetric and gynecologic sequelae such as preterm birth and low birth weight, premature rupture of membranes, amniotic fluid infection, chorioamnionitis, postpartum endometritis, pelvic inflammatory disease and early pregnancy loss (16-22). More recently BV was reported to be associated with both prevalent and incident HIV infections (2,23-25), and possibly with increased rate of mother to child HIV transmission (26).

Epidemiologic studies of early pregnancy loss are difficult as most loss occur unnoticed; clinically recognized losses represent only a fraction of the total losses. A study which determined daily human chorionic gonadotropin (hCG) levels on couples trying to conceive provided an estimate of 31% pregnancy loss. Of these, 22% were not clinically recognized and 9% were clinically recognizable (27). The

association of BV with conception and miscarriage in the first trimester of pregnancy was evaluated in a cohort study in Britain (28). Among 867 women attending an assisted conception unit, the miscarriage rate was 18.5% among women with normal flora, 23.3% among women with intermediate flora and 36.1% among women with BV. BV was associated with a two-fold risk of miscarriage during the first trimester; this was equivalent to one extra miscarriage for every 6 pregnant women with BV. Most of the miscarriages were subclinical. The most likely cause of miscarriage was postulated to be preexisting endometritis complicating implantation or early embryonic development. This study, unlike others which could only show that BV during pregnancy increases risk of late abortions (29,30), was able to determine the effect of BV on early losses because date of conception was known and vaginal samples could be obtained at the time of conception. BV did not affect overall conception rate in the study among British women (28).

Among African women, however, the effect of HIV, an infection which causes adverse reproductive outcomes such as abortions, low birth weight and increased perinatal mortality, needs careful consideration when assessing the association between BV and pregnancy loss. For example, in a population-based study in the Rakai district of Uganda (31), pregnancy prevalence was significantly reduced among HIV infected compared with uninfected women (23.5 vs 30.1 per 100 woman-years; adjusted risk ratio 0.73; 95% CI 0.57-0.93) due to lower rates of conception and increased rates of pregnancy loss. In the proposed study we will have the opportunity to evaluate the effect of BV treatment on pregnancy loss among separate groups of HIV infected and uninfected African women.

3. Treatment of bacterial vaginosis

Oral metronidazole is the treatment of choice for BV, either in a single dose or over a period of 7 days. Cure rates range from 85% to 87% when evaluated within one month, but lower cure rates with the single dose have been observed (1). Oral metronidazole, however, causes gastrointestinal discomfort and few other systemic effects (32).

Another drug for treatment of BV is clindamycin either oral or intravaginal. Clindamycin is a broad spectrum antibiotic and has activity against both aerobes and anaerobes. One potential drawback of clindamycin is that it clears lactobacilli and overgrowth of other bacteria might occur in the vagina (30,33). Due to these limitations and reports that clindamycin cream weakens condoms, we preferred use of another intravaginal agent, the metronidazole vaginal gel.

SIGNIFICANCE

We are proposing use of an intravaginal metronidazole gel in the current trial. This drug is recommended by the Centers for Disease Control (CDC) for treatment of BV (32). We will describe the active product and the placebo further in the Methods (procedures) section. This intravaginal therapy has minimal systemic effects while the cure rates are comparable to the oral regimen. The short regimen (5 days) will encourage compliance. Additionally, since use of traditional vaginal agents is common among Malawian women (see Preliminary Studies), use of intravaginal gel will be acceptable, woman controlled, and does not require partner cooperation. In addition to its reported effectiveness in treating BV, other infections such as trichomonas could be reduced. The study will provide evidence-based information about the effect of a simple treatment intervention on adverse reproductive outcomes associated with BV (e.g., pregnancy loss and pelvic inflammatory disease) among HIV infected and uninfected African women. The research questions we are addressing concerning BV and its treatment using metronidazole intravaginal gel are important, and to the best of our knowledge have not been studied in sub-Saharan Africa where HIV/STDs and adverse reproductive outcomes are common. Simple interventions that can be applied at a wider scale are

urgently needed to improve the health of mothers in developing countries. In Malawi, infectious complications represent a major cause of maternal morbidity and mortality. The estimated maternal mortality ratio ranges between 450-1452 per 100,000 live births (Mtimavalye, Unpublished report, 1999). This study (designed to enroll 832 HIV infected and 832 HIV uninfected women, equally randomized to treatment or placebo arms) complements our on-going vaginal microbicide and perinatal research trials in Malawi. This study maximally utilizes existing infra-structure, and enrolls women who are "passively" participating in two on-going perinatal trials where only the baby is of primary interest for the outcome of the on-going trials (see Study Population and Recruitment; Section D.1.).

C. PRELIMINARY STUDIES
THE EXISTING COLLABORATION IN MALAWI

The Republic of Malawi is landlocked and shares borders with Mozambique, Tanzania and Zambia (Appendix 1: Map). The capital is Lilongwe in the central region and the biggest commercial center is Blantyre in the southern region. Malawi achieved independence from Britain in 1964 and since then has had a stable political system. With a population of approximately 12 million, the country is one of the most densely populated in Africa. According to the Demographic and Health Survey in 1993, the infant and child mortality rates were 134 and 234 per 1000 live births, respectively. Currently, it is estimated that about one million people are infected with HIV, and approximately, 200,000 people have died from AIDS. The major mode of HIV transmission is heterosexual, accounting for approximately 90% of infections.

The Johns Hopkins University (JHU) Research Project in Malawi. The JHU Research Project in Blantyre, Malawi has been conducting research since 1988. The Project started in collaboration with the Malawi Ministry of Health (MOH); the College of Medicine (COM) joined this collaboration when it was founded in 1991. This long-standing interaction between JHU, MOH and COM has helped to establish a strong trust-worthy and stable relationship with the MOH, COM, and the local co-investigators. Currently, more than seven COM faculty members are participating in our on going research. JHU has provided several advanced training opportunities (doctoral, masters, short term) for Malawian candidates in public health, clinical medicine and medical informatics.

The JHU Research Project has carried-out studies on HIV and STDs in a systematic manner (Tables 1 and 2). The initial JHU studies were descriptive and observational where the prevalence and incidence of HIV/STDs, risk factors for HIV infection, rates of mother to child transmission of HIV, and the impact of HIV/AIDS on mothers and children were investigated. Based on this information, clinical trials were conducted in the fields of perinatal, microbicide and nutritional supplementation. This logical sequence of activities was well received by policy makers in Malawi as well as by the international scientific community. The MOH and COM have continuously facilitated our work in the country. This collaborative research team from JHU, COM and MOH has consistently hoped to develop and support research needs of mothers and children in Malawi. This proposal fulfills this need.

Table 1. Observational studies conducted by the JHU-COM Research Project in Malawi, 1989-99*

Year	# Screened (enrolled)	Objectives of the Study
1989-1990	6,603 (1,366 mothers)	● Determine prevalence/incidence, risk factors and impact of STDS and HIV.
1993	2,471 (1,100 mothers)	● Study HIV seroconversion rates/risk factors.
1994-99¶	1,690 (1,690 men)	● Determine HIV incidence among men working in a sugar estate (SUCOMA).
1995-97	2,013 (1,633 mothers)	● Study risk factors associated with STD/HIV.
1995-97	853 (853 babies)	● Study HIV transmission due to breastfeeding.
1996	1,200 (1,020 mothers)	● Determine the effect of HIV infection on maternal morbidity and mortality.
1999-02	150 (800** mothers)	● Study the epidemiology and microbiology of mastitis and its association with MTCT.

Table 2. Intervention studies conducted by the JHU-COM Research Project in Malawi, 1989-99*

Year	# Screened (enrolled)	Objectives of the Study
1994-95	6,964 (6,964 women)	● Effect of washing the birth canal with chlorhexidine to prevent MTCT of HIV and other infections.
1995-98	3,949 (985 women)	● Determine the effect of prenatal vitamin A supplemention on MTCT of HIV and birth outcomes.
1997-98	159 (20 women)	● Phase I safety study of BufferGel microbicide.
1998-02†	139 (60 women)	● Determine the efficacy of the microbicide N–9 in reducing incident HIV and STDs.
1999-03‡	Has started (adults)	● Determine daily supplementation of vitamin A effect and TB therapy on outcome of HIV and pulmonary TB.
1998-02†	To start (3,100** women)	● Determine efficacy of chorioamnionitis antibiotics treatment in reducing MTCT of HIV & birth outcomes.
1999-03	200 (1,800** women)	● Determine effect of a very short antiviral regimen of AZT & NVP on MTCT of HIV.

* All studies conducted at the QECH in Blantyre, if not indicated.
** Required sample.
† Multi-site clinical trial conducted in Blantyre (QECH) and Lilongwe, Malawi.
‡ Conducted at the Zomba Central Hospital in the southern region.

STUDIES ON BACTERIAL VAGINOSIS (BV) AND STDS AMONG PREGNANT AND POSTNATAL WOMEN IN MALAWI

Because HIV prevalence is high in Malawi, our studies have been stratified by HIV status of the women and children. We have enrolled appropriate samples of uninfected women and children in these studies to maintain confidentiality and to provide additional comparison groups.

1. Bacterial vaginosis

Prevalence among pregnant and postnatal women. The prevalence of BV and other forms of disturbance of vaginal flora are common among Malawian women. This is consistent with reports from other African countries (2,3). As shown in Table 3, among two large cohorts of pregnant women screened in 1990 and 1993, respectively, only 11% of women had normal vaginal flora, about 59% had a mild to moderate disturbance and 30% had a severe disturbance (i.e., BV) (34). We used clinical criteria to define BV. Table 4 shows that BV, trichomoniasis and candidiasis each accounted for rates of approximately 14 to 16 per 100 person-visits among women we followed postnatally in Malawi (24).

Table 3. Prevalence of disturbances of vaginal flora among pregnant women surveyed in 1990 and 1993, Blantyre, Malawi.

Disturbance of vaginal flora*	N=9126 (1990/1993)
Normal	10.7%
Mild (1 criterion)	38.3%
Moderate (2 criteria)	21.0%
Severe (BV: 3 or more criteria)	30.0%

* Based on the following clinical criteria: vaginal pH>4.5, vaginal discharge, presence of clue cells≥20%, and a positive amine (Whiff) test.

Table 4. Frequency of bacterial vaginosis and STDs among postpartum women in Blantyre.

Disease	Person visits	Event-visits	Rate/100 person visits	95% CI
Bacterial vaginosis	4479	736	16.43	15.20-17.58
Gonorrhea	4130	44	1.07	0.75-1.39
Trichomoniasis	4015	567	14.12	12.70-15.50
Syphilis	4905	128	2.61	1.82-3.40
Candidiasis	4012	561	13.98	12.78-15.14

Association of BV with adverse reproductive outcomes. There were consistent associations between disturbances of vaginal flora and low birth weight, reduced mean birth weight, preterm birth (<37 weeks gestation) and higher neonatal mortality. Table 5 shows that there was a statistically significant trend of associations between disturbance of vaginal flora and these outcomes: adverse outcomes were lowest among women with normal vaginal flora and highest among women with BV - severe disturbance.

Table 5. Association of disturbance of vaginal flora (based on clinical criteria) with adverse reproductive outcomes among women enrolled during pregnancy in 1990.

	Normal N=124	Mild N=441	Moderate N=315	Severe N=473	P-value*
Mean birth weight (g)	2977	2948	2908	2820	0.001
LBW** (<2,500g) %	9.7	14.3	18.1	20.3	<0.003
Gestational age <37wks %	14.5	17.0	18.8	23.5	<0.005
Neonatal deaths*** %	3.1(N=97)	4.2(N=335)	8.0(N=251)	8.0(N=324)	0.012
Adverse outcomes combined ¶	19.2	23.9	27.6	30.4	0.003

* X^2 trend for LBW, gestational age <37 weeks and neonatal deaths; ANOVA for mean birth weight comparisons.
** Low birth weight.
*** Figures in brackets are actual denominators.
¶ Women having either LBW, short gestational age (<37 weeks) or neonatal deaths.

BV association with HIV infection. Increasing severity of disturbance of vaginal flora was significantly associated with prevalent HIV infection (Table 6). This trend persisted after adjusting for concurrent STDs, sexual activity, and socioeconomic factors. As expected, STDs such as syphilis, gonorrhea and trichomoniasis were significantly associated with prevalent HIV infection (34). More importantly, in our longitudinal studies, BV was significantly associated with acquisition of HIV during pregnancy and postpartum. Among 1196 HIV seronegative women who were followed antenatally for a median of 3.4 months, 27 women seroconverted by time of delivery. Postnatally, 97 seroconversions occurred among 1169 seronegative women who were followed for a median of 2.5 years. BV was significantly associated with antenatal HIV seroconversion (adjusted odds ratio=3.7) and postnatal HIV seroconversion (adjusted rate ratio=2.3). There was a significant trend of increased risk of HIV seroconversion with increasing severity of vaginal disturbance among both antenatal and postnatal women. The approximate attributable risk of BV alone was 23% for antenatal HIV seroconversions and 14% for postnatal seroconversions (24). In addition to BV, this study showed that syphilis, gonorrhea and trichomoniasis were independently associated with HIV seroconversion.

Table 6. Prevalence of HIV among pregnant women with disturbance of vaginal flora, 1990 and 1993 cohorts, Blantyre, Malawi.

Disturbance of vaginal flora	1990 (N=6677)	1993 (N=2449)
Overall	22.5%	30.0%
Normal	10.1%	18.1%
Mild	17.0%	24.4%
Moderate	24.9%	33.5%
Severe	30.8%	43.9%
P-value*	<0.0001	<0.0001

* X^2 trend test.

BV and mother to child transmission (MTCT) of HIV. A preliminary analysis of the rate of MTCT of HIV among women who had BV during pregnancy showed that the rate of HIV transmission was 14% among women who had normal vaginal flora and 28% among women with BV (a two-fold increase) (26). The findings suggest that BV could increase perinatal transmission. This is particularly important since BV and other genital tract infections were associated with risk of HIV acquisition

during pregnancy (24).

2. Sexually transmitted diseases (STDs) among pregnant and postpartum women

Prevalence of STDs. The prevalence of STDs among pregnant and postpartum women has been assessed in several cohorts. Table 7 shows that STDs are common among women of childbearing age in Malawi. The prevalence of STDs was particularly high among HIV infected women (35). Recently, there has been a declining trend in STDs without substantial increases in the rate of condom use over time (35), possibly due to improved STD diagnosis and treatment, or reduced risk behaviors.

Table 7. Trends of STDs among pregnant women surveyed at QECH, Blantyre, Malawi

Genital infection	1990 N=6603	1993 N=2161	1995 N=808	1996 N=829	P-value*
	%	%	%	%	
Syphilis	13.4	12.2	12.1	11.1	0.027
Trichomoniasis	32.5	28.6	23.8		0.000
Gonorrhea	4.9	2.5	2.5		0.000
Genital warts	4.8	3.1	2.5		0.000
Genital ulcers	6.8	6.7	3.4		0.004

* χ^2 test for trend

Incidence of STDs. The cumulative incidence of gonorrhea, trichomoniasis, genital ulcers and genital warts (based on laboratory and clinical data) among a cohort of 644 HIV seropositive and 677 HIV seronegative women during the period November 1989 to November 1992 was significantly higher in HIV seropositive than in HIV seronegative women. The rates of these infections among HIV infected and uninfected women were 19.8% vs 7.9% for gonorrhea ($p<0.001$), 51.3% vs 35.6% for trichomoniasis ($p<0.009$), 26.9% vs 9.3% for genital ulcers ($p<0.001$), and 23.6% vs 13.6% for genital warts ($p=0.030$). The higher rate of new STDs in HIV infected women may represent increased susceptibility, higher risky behavior, or in the case of genital warts, compromised immune status of the woman.

HIV prevalence and incidence. The seroprevalence of HIV among pregnant women attending their first antenatal visit at the Queen Elizabeth Central Hospital (QECH) in Blantyre rose from 2.0% in 1985 to 31.0% in 1998, about a sixteen-fold increase (35). Among two cohorts of women of childbearing age recruited in 1990 and 1993, the incidence of HIV was high (overall incidence of 4.2 per 100 person years). As shown in Table 8, the HIV incidence was highest among young women below 20 years of age and progressively decreased with increase in age (24,35). The rate of seroconversion was significantly higher during pregnancy compared with postpartum rates; Table 9. The high rates during pregnancy could be due to increased estrogen and progesteron, and local cervical factors (e.g., increased vascularity, exudation and ectopy (24). HIV acquisition during pregnancy and the accompanying initial high viremia could potentially increase HIV transmission from mother to infant.

Of interest to note is that the rate of HIV acquisition after delivery is highest in the early postnatal period (during first 6 months) (35). Data from our qualitative studies suggest that for cultural reasons sexual activity is low in late pregnancy and early postpartum. As described in other African countries (36), it is likely that men are sexually active outside their regular partnerships during this period of abstinence with their wives. Upon resumption of sexual activity with their regular partners, however, these women are at increased risk of sexually transmitted infections. Therefore, interventions such as intravaginal antibiotics and microbicides to treat or prevent genital tract

infections are needed to provide protection when sexual activity is resumed after delivery.

Table 8. Postnatal age-specific HIV incidence among women recruited in 1990 & 1993

Age group	New HIV infections	Person years of follow-up	Incidence rate (per 100 person yrs)	95% CI
< 20	30	502	5.98	5.84-8.12
20-24	32	699	4.58	2.99-6.17
25-29	21	496	4.23	2.42-6.04
30-34	12	343	3.50	1.60-5.40
35+	2	262	0.76	0.01-1.81

Table 9. HIV Incidence in cohorts of pregnant women recruited in 1990 and 1993

	New HIV infections	Person-years	Incidence rate/100 p-y	95% CI
Antenatal¶	27	338	7.9	4.9-11.0
Postnatal§	97	2,684	3.6	2.9-4.3

¶ 1,196 HIV seronegative women enrolled at first antenatal visit and followed for a median of 3.4 months during pregnancy. § Follow-up of 1,169 HIV seronegative women was continued for a median duration of 2.5 years after delivery.

3. Selected risk factors studied among women of reproductive age in Malawi

Use of traditional vaginal agents. Use of traditional vaginal agents (desiccants, leaves, stones, etc.) for treatment of itching and vaginal discharge or for tightening purposes were investigated. Forty five percent of women attending the antenatal clinic at QECH reported self-treatment of vaginal discharge and itching and 13% (886) reported using vaginal agents for tightening of the vaginal wall. Use of intra-vaginal agents for treatment was slightly higher among HIV infected women than among uninfected women [17% vs 14%; OR=1.3, 95% CI 1.05-1.57, p=0.01]. There was no difference in HIV status when these agents were used for vaginal tightening. In multivariate analysis, vaginal agent use for treatment was independently associated with HIV seropositivity. The association of HIV infection with vaginal agents for self-treatment, but not for tightening, suggests that STDs may play a role or that vaginal agents are used differently for the two purposes (37). In addition to a small increased risk of HIV infection associated with use of vaginal agent, traditional agents may influence condom effectiveness or use of vaginal microbicides.

Condom use. Rates of condom use are low in Malawi. The trends of condom use are summarized in Table 10. Our data show that there has not been substantial changes in the rates of condom use (life-time use). Among women who were repeatedly counseled on condom use and followed every 6 months for 2 years, condom use was inconsistent and over-reported especially among HIV infected women (38). Unwillingness of male partners to use condoms (the best barrier available), and inability of women to negotiate sex with their partners, strongly justifies the search for alternative simple treatment measures for genital tract infections (such as intravaginal antibiotics).

Table 10. Trends of condom use among women surveyed at QECH, Blantyre, Malawi

	1990			1993			1995		
	N	%	95% CI	N	%	95% CI	N	%	95% CI
All women	6603	5.6	5.1-6.2	2103	17.5	15.9-19.1		4.9	3.4-6.4
HIV pos. women	1502	7.1	5.8-8.4	616	22.2	18.9-25.5		5.0	3.3-6.6
HIV neg. women	5101	5.2	4.6-5.8	1487	15.6	13.8-17.4		4.1	0.2-8.0

4. Impact of STDs and maternal factors on child mortality and morbidity in Malawi

Child mortality. The overall national infant and child mortality rates are high in Malawi. The substantial differences in mortality between HIV infected and uninfected children are generally attributed to HIV infection. For example, in the first cohort of children we enrolled in 1990, the infant mortality of children born to HIV seropositive mothers was 223 per 1000 compared with 68 per 1000 among children born to HIV seronegative mothers (39). Both child mortality and spontaneous abortions in a prior pregnancy were more frequent among HIV positive than among HIV negative mothers (40). In addition to HIV, these adverse outcomes were associated with history of syphilis and other STDs. Among 702 children with confirmed HIV status based on PCR at 6 weeks of age and enrolled in a cohort study in 1997, the second and third year mortality rate per 1000 person years of observation was 339.3 among HIV infected children; 46.3 among uninfected children born to seropositive mothers; and 35.7 among children born to uninfected mothers. These mortality differentials between HIV infected and uninfected children persisted after adjusting for several risk factors. The major causes of death among infected children (N=52) were wasting and respiratory conditions (41). Among a subgroup of HIV infected children, viral load and CD4 values were the main predictors of mortality (42).

Child morbidity. Among 808 children with confirmed HIV status and followed prospectively for more than two years from late infancy to age 3 years, age adjusted morbidity rates were significantly higher among HIV infected compared with uninfected children. HIV infected children were more immunosuppressed and their longitudinal growth declined starting at an earlier age compared with uninfected children. By three years of age, 89% of the infected children died, 10% were in category B or C (CDC Clinical Classification (43)), and only about 1% of the children were without HIV symptoms. A high frequency of diseases, and rapid progression from asymptomatic or symptomatic HIV disease to death were observed in this cohort despite high childhood immunization coverage in all groups of children (44). Because immunization for conventional childhood diseases was high in our previous cohorts, a study of the natural history of other conditions not included in the current vaccination program is important. This can be achieved in the proposed study by regular follow-up of children and monitoring of their morbidity and mortality. There is a critical need for studies to identify etiologic agents. Knowledge of these factors may assist in providing appropriate clinical care and designing potential interventions.

Maternal death and child survival. A great concern in sub-Saharan Africa is the increased incidence of children becoming orphans as the HIV epidemic spreads. We examined data collected from cohorts of women enrolled in our studies during 1990 and 1993 (2829 women enrolled) to determine if death of a mother leads to an excess in child mortality (45). As expected, death of a mother was significantly associated with child mortality after adjusting for several risk factors (odds ratio 3.25; $p<0.001$). Inadequate nutrition due to interruption of breastfeeding, insufficient child care or increased mother to child transmission of HIV in late maternal HIV disease (if the woman is infected) are factors most likely involved in this excess child mortality.

Mother to child transmission (MTCT) of HIV. Accurate estimates were obtained in two clinical trials conducted in 1994 and 1996 using repeat PCR testing. In the birth canal cleansing intervention study (46) the HIV MTCT rate was 28% in babies tested at 6 and 12 weeks of age, and in the vitamin A prenatal supplementation trial the MTCT rate at 6 weeks was 27% (47).

Breastmilk and HIV transmission. HIV uninfected babies were prospectively followed to determine the magnitude of HIV transmission attributed to breastfeeding. Forty seven (7.0%) of 672 children became HIV infected while breastfeeding, but none after breastfeeding had stopped. The cumulative infection rate while breastfeeding, from month one to end of months 5, 11, 17, and 23, was 3.5%, 7.0%, 8.9%, and 10.3%, respectively. Inicidence per month was 0.7% during age 1-5 months, 0.6% during age 6-11 months, and 0.3% during age 12 to 17 months (p=0.01 for trend) (48). The data suggest that the risk of HIV infection is highest in the early months of breastfeeding. This high early transmission risk compounds difficulties in formulating appropriate breastfeeding policies in areas where the prevalence of HIV is high and breasfeeding is universal.
Maternal nutrition. Among 338 HIV positive mothers whose infant's HIV status was known, mean vitamin A concentration in 74 mothers who transmitted HIV to their infants was lower than that in 264 mothers who did not transmit HIV to their infants (0.86 umol/L vs 1.07 umol/L, p<0.0001). The rates of transmission were inversely related to the level of vitamin A in the mother: the lower the serum vitamin A concentration, the higher was the transmission rate (49).

5. Perinatal studies conducted by the Johns Hopkins University Research Project in Malawi
The chlorhexidine vaginal cleansing trial. This trial was conducted at the QECH in Blantyre in 1994/95. We enrolled 3,635 women giving birth to 3,743 babies in the intervention phase and 3,330 women giving birth to 3,417 babies in the non-intervention phase. Women delivering during the intervention phase had manual wiping of the entire birth canal including the external genitalia with a cotton swab soaked in 0.25% chlorhexidine solution prior to every vaginal examination. Babies born during the intervention phase were also wiped with chlorhexidine-soaked pads immediately after delivery. The intervention had no significant impact on HIV transmission rates (27% in 505 intervention women and 28% in 477 control women; p=0.74), except when membranes were ruptured for >4 hours before delivery (25% transmission rate in the intervention group vs 39% in the control group; p=0.02) (46). It is plausible that the birth canal washing when the fetal membranes were prematurely ruptured has resulted in reduction of ascending HIV infection. Inability to demonstrate a significant impact of this simple intervention could be due to several factors. A 0.25% chlorhexidine solution could be ineffective due to low virucidal concentration, or the presence of the membranes and fetal parts might limit efficient wiping of the entire birth canal.

However, cleansing of the birth canal with this simple antiseptic significantly reduced early neonatal and maternal postpartum infections (50). Among infants born in the intervention phase, there were reductions in overall neonatal admissions (16.9% vs 19.3%, p<0.01), neonatal sepsis admissions (7.7 vs 17.9 per 1000 live births, p<0.0002), overall neonatal mortality (28.6 vs 36.9 per 1000 live births, p<0.06), and mortality due to infectious causes (2.4 vs 7.3 per 1000 live births, p<0.005). Overall, early neonatal mortality decreased by 25% during the month when the intervention was introduced compared to that of the month preceding the intervention (31.6 vs 42.1 per 1000 live births). Among mothers receiving the intervention, reductions were observed in delivery-related postpartum admissions (29.4 vs 40.2 per 1000 deliveries, p<0.02), admissions due to postpartum infections (1.7 vs 5.1 per 1000 deliveries, p=0.02), and duration of median hospitalization (Wilcoxon p=0.008).
The vitamin A supplementation clinical trial. A randomized placebo-controlled clinical trial was conducted at QECH in Blantyre to determine the effect of vitamin A supplementation during pregnancy on mother to child transmission of HIV. This trial was based on our earlier finding that women who were deficient in serum vitamin A were more likely to transmit HIV to their newborns (49). Preliminary analysis shows that vitamin A supplementation had no significant impact on HIV MTCT (47). Supplementation with vitamin A, however, had significant beneficial effects on birth weight and gestational age (Semba, Unpublished data).
The mastitis study. This observational study is conducted at QECH in Blantyre to determine the point

prevalence of mastitis, to characterize the main microbiological pathogens involved in mastitis, and to determine the optimal antibiotic treatment for mastitis. The study is based on our earlier findings which suggested that mastitis could increase the risk of MTCT of HIV among breastfeeding women (47). This study will determine the basic epidemiology, microbiology, and treatment of mastitis and should provide insight into future strategies to reduce mastitis among breastfeeding women.
The chorioamnionitis treatment trial (HIVNET 024). This on-going study is a randomized, double blinded, controlled phase III trial of antibiotics to prevent chorioamnionitis-associated perinatal HIV transmission. In a two arm design, half of the participants (sample size of 1,600 HIV positive and a sample of HIV negative women to maintain confidentiality) will receive two courses of oral antibiotics, and the other half will receive two courses of an oral placebo. The intervention attempts to reduce perinatal HIV transmission through a low cost antenatal and intrapartum antibacterial therapy. Antibiotic treatment is provided antenatally around 20-24 weeks of gestation for treatment of possible chronic chorioamnionitis, and repeated at time of delivery/intrapartum for treatment of potential acute chorioamnionitis. The antibiotics used are oral erythromycin and metronidazole antenatally, and oral ampicillin and metronidazole at delivery. Although the effectiveness of this antibiotic treatment approach in reducing HIV perinatal transmission is yet to be determined, it is anticipated (from prior studies) that this approach could have secondary benefits such as lowering of preterm birth rate, improving the birth weight of the newborn, and reducing maternal/infant morbidity and mortality. Based on recent results from Uganda on effectiveness of Nevirapine (51), all women and children participating in this trial will receive a single dose of Nevirapine. This trial is supported by the Division of AIDS, NIAID, NIH.

Women giving birth in this study will represent a major recruitment pool for the current intravaginal gel treatment trial. Only the baby is actively followed in the chorioamnionitis antibiotic treatment trial. Women are not active participants; they routinely attend the study postnatal clinic to bring the child for scheduled visits or to receive medical care when it is needed. Women who were screened during pregnancy and were HIV negative will also be eligible for enrollment after delivery.

Zidovudine (AZT)/Nevirapine (NVP) at birth study (NVAZ Trial). This trial attempts to determine, in a traditionally breast-feeding community in Malawi, if a short prophylactic regimen of oral NVP or NVP plus AZT given only to the baby reduces the rate of HIV MTCT. This study assesses the effectiveness of NVP or NVP plus AZT in a "real-life" situation where some women will be attending the labor room late and there will be no adequate time to counsel, HIV test, and administer the drug NVP to the mother. Children of mothers who receive or did not receive NVP before delivery (based on their time of admission to the labor room) will be randomized to receive either a single dose of oral NVP, or receive the same dose of NVP in addition to oral AZT. This trial is supported by the Fogarty International Center and the Doris Duke Charitable Foundation. About 2,200 women will be enrolled in this trial. To-date, about 800 women have been successfully enrolled.

Similar to the chorioamnionitis antibiotic treatment clinical trial above (HIVNET 024), women giving birth in this NVAZ trial represent another major recruitment population for the current intravaginal gel treatment trial. Only the baby is actively followed in the NVAZ trial. Women are not active participants; they routinely attend the study clinic to bring the child for scheduled visits or to receive medical care when it is needed. Therefore, these women will be eligible postnatally for recruitment. Women who were screened at delivery and found HIV negative will also be eligible for enrollment after delivery.

6. Vaginal microbicide studies conducted by the Johns Hopkins University Project in Malawi
Due to gender imbalances and cultural factors in several African societies, women have no power to negotiate use of barriers with their male partners. Therefore, the development of a preventive measure such as a female microbicide which women can control is urgently needed to prevent the acquisition

of HIV and STDs. This is particularly important in Malawi where, as noted earlier, the incidence of HIV is high among women of childbearing age (24) and the rate of the male condom use is very low (see Table 10) and is inconsistent (38). A HIVNET supported multi-site phase 1 trial of the microbicide BufferGel was conducted in Malawi to assess the local toxicity and acceptability of BufferGel for vaginal use. Preliminary results of this study show that the product was safe and acceptability was high (52). We have aslo completed a pilot phase of a 100 mg N-9 gel (Conceptrol). A condom promotion phase to provide counseling and intensive condom promotion is currently underway. The proposed study optimally complements these activities and, if successful, could provide a practical measure to reduce genital tract infections.

D. RESEARCH DESIGN AND METHODS

D. 1. RESEARCH DESIGN TO ACHIEVE SPECIFIC AIMS

Research design. This is a randomized, double masked, placebo-controlled clinical trial. The study will have two arms: randomly assigned women will receive either an intravaginal gel with the active product or an intravaginal gel without the product (placebo). We have opted to use a mass treatment approach because the overall prevalence of disturbances of vaginal flora is high among Malawian women of childbearing age (89%; reference 34).

Study population. The study population will consist of women currently screened for enrollment in two perinatal clinical trials: a) the chorioamnionitis antibiotic treatment trial (HIVNET 024) and, b) the Zidovudine/Nevirapine at birth study (NVAZ trial). These women are screened and enrolled from several antenatal clinics and labor rooms of health institutions in Blantyre and Zomba, Southern Malawi. In the on-going trials, all women attending these health centers and hospitals are consented and screened for HIV. HIV positive women are offered study-specific treatments (either antibiotics (HIVNET 024) or antiretrovirals (NVP/AZT)) to prevent mother-to child transmission of HIV. Babies are followed every three months from birth to 12 months. Mothers are "passive" participants; they only receive medical care, if needed.

Recruitment. Participants will be recruited postnatally into the proposed study from women who had already been screened for HIV either antenatally in the chorioamnionitis treatment study or at delivery in the NVAZ study. HIV infected women and HIV uninfected women who were screened in these trials, but were not eligible for enrollment, will be recruited into the current trial. These women will be counseled and consented after delivery, before discharge from the hospital, to enroll in the proposed new study - the intravaginal gel trial. Women who agree to enrol will be scheduled to return for their fist follow-up visit at 3 months. These women will be re-counseled to confirm their desire to be part of the current trial at the 3-month postnatal visit. Study participants will subsequently return for postnatal follow-up visits at 6, 9 and 12 months as shown in Figure 1. The total duration of the study with product use is 12 months; i.e., four courses of treatment will be used during the trail. The postnatal maternal visit schedules are consistent with the child's visits (for those participating in the perinatal trials).

Figure 1. Recruitment and follow-up.

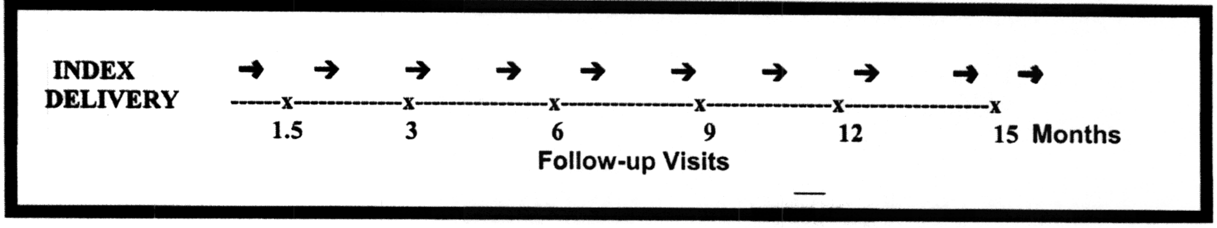

Women will be randomized at the three months postnatal visit and issued the intervention (either metronidazole gel or placebo). The timing of randomization and initiation of therapy approximately coincides with resumption of sexual activity in Malawi.

Recruitment of women from the on-going NVAZ and chorioamnionitis perinatal trial population has several advantages: a) is cost-effective because the existing infra-structure of clinic personnel, counseling and testing, and the same child/mother follow-up arrangements will be utilized; b) participating women might benefit from the trial; c) involvement of women in on-going research for the baby could increase rates of retention in these studies because women will become active participants; and d) enrolling both HIV infected and infected women in these clinics will maintain

confidentiality of participants and eliminate potential discrimination.

All women will be tested for disturbance of vaginal flora and syphilis at the first visit (3 months postnatally) and at each subsequent visit. To determine if this treatment reduces T. *vaginalis* infection, vaginal wet mounts obtained from all women (HIV negative and positive) will be examined at each visit. Urine samples will be tested at 6, 9 and 12 months postnatal visits to confirm conception and estimate rates of early pregnancy loss. Information on the last menstrual period (LMP) and history of bleeding (or spotting) will also be collected at each visit. Abortions and stillbirths will be observed and documented throughout the follow-up period. For the secondary objective (to determine clinical and behavioral factors which facilitate or impede the long-term use of metronidazole vaginal gel in a community where use of traditional vaginal agents is common) qualitative and quantitative clinical and behavioral data will be longitudinally collected postnatally to compare side-effects, compliance and acceptability between women who use the active product or the placebo. The information collected at each visit will pertain to events occurring during the past three months; i.e., most recent events. HIV negative women will be tested to confirm HIV seroconvesion. HIV serology will be performed on the same blood sample which is used for syphilis testing.

D. 2. STUDY PROCEDURES

We will pilot test appropriate study procedures and train study personnel at the study sites, and perform revisions and adjustments before the start of the main trial.

D. 2. a. Counseling and consenting. Women will be counseled about the study after delivery. Counseling will include details of the study and the need for STD/HIV testing. In the on-going NVAZ perinatal trial, two separate written consents are obtained, one to perform HIV testing, and the other to enroll in the perinatal trial (if HIV infected). We will obtain an additional enrollment written consent after delivery to request a woman's participation in the new study at the 3 months visit at time of randomization.

D. 2. b. Inclusion and exclusion criteria. The inclusion criteria are: ability and willingness to give a written informed consent; willingness to return for follow-up visits after delivery; willingness to use the product as instructed; willingness to provide specimens for pregnancy and STD tests (including HIV for seronegative women); and resident of the study area. Exclusion criteria at enrollment will include inability to provide informed consent, woman's desire to move from the study area, and refusal of any of the inclusion criteria.

D. 2. c. Enrollment and randomization. Women will be requested to enroll after they are informed of their HIV status and the need for regular visits throughout the duration of the study. Woman who sign the enrollment consent form will be randomized and provided the intravaginal gel treatment. A computer generated randomization list will be used to assign women to the appropriate treatment arms. Randomization will be in blocks of eight with four randomly chosen women of each block receiving treatment.

D. 2. d. HIV testing. In this study, all women will be appropriately counseled, consented, and subsequently tested for HIV before final enrollment into the study. Women who successfully enroll and are HIV negative will be tested at the 3, 6 and 12 months postnatal visits (unless a woman seroconverts). Women who are positive on repeat ELISA testing (using for example, Genetic systems and Wellcozyme tests) will be identified as infected. Western blot tests will be used for confirmation of borderline ELISA results and to confirm all cases of HIV seroconversions.

D. 2. e. Syphilis testing. Syphilis testing is mandatory during pregnancy in Malawi. After this initial syphilis testing, women will be followed longitudinally and screened for syphilis every six months

until study closure. Syphilis treatment will be provided at no cost for the woman and her partner if the test results are positive at any visit. Screening for syphilis will be performed using rapid plasma reagin (RPR) test and confirmed using *treponema pallidum* hemagglutination assay (TPHA) test.

D. 2. f. Disturbance of vaginal flora testing. The diagnosis of disturbance of vaginal flora and BV will be based on the presence of clinical criteria and will be confirmed by microscopic examination of vaginal smears (53) at each visit. The clinical definition is based on the following criteria: a) a vaginal pH >4.5 (measured by pH paper on a vaginal swab obtained from lateral and posterior fornices); b) an increased homogeneous vaginal discharge; c) presence of clue cells in ≥20% of vaginal epithelial cells (detected by mixing vaginal fluid with a drop of normal saline on a slide and examining under high power magnification); and d) a positive amine or Whiff test (performed by mixing a few drops of 10% potassium hydroxide with vaginal fluid). As we described in our previous studies (24,34), a woman with none of the clinical criteria will be described as having normal flora, only one criterion will be described as having mild disturbance, two criteria as having moderate disturbance, and three or more criteria as having severe disturbance or BV. This definition of BV is consistent with the conventional method based on Amsel's (54) criteria and could be equivalent to a score of 7-10 based on the Nugent vaginal smear gram stain scoring method (53).

D. 2. g. Pregnancy testing. We will use a highly sensitive commercial kit to detect hCG levels in a urine sample to detect pregnancy. Ultrasound to confirm pregnancy is not routinely available in Malawi and will not be used in this study; we will rely on the date of last menstrual period and pregnancy testing.

D. 2. h. Other laboratory tests. A vaginal wet mount will be examined microscopically at each visit to detect motile trichomonads and candida albicans (hyphae and buds). Wet mount examination to detect trichomonads is not highly sensitive; therefore, vaginal swabs will be archived, and PCR or culture will be considered. These tests, however, are expensive and are not performed locally in Malawi. Overgrowth of candida is a possibility with use of intravaginal antibiotics (1). We will test for candida at each visit. We will not routinely test all women for gonococcal or chlamydial infections because the rates are low (see Table 7 for prevalence of gonorrhea). A recent N-9 pilot study found chlamydia in less than 1% among women of reproductive age at two sites in Malawi. Maternal and infant hemoglobin will be performed using a finger or heal prick blood spot by a portable (Hemocue) machine. The hemoglobin results will be immediately available.

D.2. i. Management of STD and BV cases. As we indicated earlier, women (and their partners) with syphilis will be provided treatment at no cost. We will follow the Malawi recommendations and provide syndromic treatment for other STDs. Treatment of BV is not included in this WHO recommended approach of syndromic management. Women with discharge, however, are either treated for trichomonas infection (using oral metronidazole) or for vulvovaginal candida infection. Since oral metronidazole might influence rates of BV, details of treatment (type of drug, dose and duration) will be documented at each visit on a case report form. We will not deny a participant any medication recommended by a clinician. The data analysis will take into account concomitant medications.

D. 2. j. Physical and anthropometric examination. Trained nurse-midwives will perform physical examinations of the mother at each visit based on structured procedures documented in the manual of operations. Standard scales for measurement of weight and height will be used. The staff will be trained and adequately supervised, and machines will be regularly recalibrated.

D. 2. k. Study questionnaires. Trained interviewers and nurse midwives will complete structured questionnaires. A baseline demographic and clinical questionnaire will be completed at enrollment and updated at follow-up visits. A delivery examination form will be completed before discharge of

the woman from the hospital. Baseline behavioral factors such as number of sexual partners, sexual practices, and use of traditional vaginal agents and condoms will be obtained at enrollment and updated at each visit. Maternal morbidity and mortality information will be obtained using structured questionnaires.

D.2.l. Acceptability. Consistent use of intravaginal metronidazole gel as prescribed (once daily, 5 consecutive days every 3 months) among the study population is necessary to assess gel safety and efficacy, evaluate the overall efficacy of the intervention, and to study factors which could influence program implementation if the intervention proves to be successful. Compliance, however, requires acceptability of the product by the study participants. To ascertain product acceptability, we will use qualitative and quantitative techniques to gather information about the product's sensory characteristics, packaging, method of administration, and other behavioral factors related to product use. Information on social and cultural factors that may influence product use will also be collected. A questionnaire will be administered at enrollment and follow-up visits to assess factors associated with product use and related perceptions or behaviors. In addition, exit focus groups consisting of 4-6 participants will be conducted at enrollment and every six months thereafter. Focus groups with 4-6 male partners of the participants will also be conducted at 6 and 12 months to elicit information about product use, perceptions of the gel and related contextual factors from the partners' perspective. Investigated dimensions of product use that may affect full compliance include specific perceptions of side effects of use (smell, discharge, embarrassment, mess, etc.) and perceptions of partner reactions. Sociodemographic and behavioral correlates of product use, including age, duration of sexual relationship, douching, finger cleansing and tampon preference, will be assessed.

D.2.m. Measurement of compliance. Women will be issued a tube (containing either the active product or placebo) with a specific identification number at each visit. To monitor compliance, women will be requested to return the empty tube at each follow-up visit. The identification number on the tube will be checked.

D.2.n. Follow-up visits and measures to reduce loss to follow-up. In this study women will return for follow-up visits every three months after the first visit at 6 weeks following delivery. At every visit women will receive a complete physical exam, specimens will be obtained, and questionnaires will be completed. In recent cohorts of women and children we have studied, the follow-up rates have been excellent. In an observational study to determine the magnitude of HIV transmission due to breast feeding, 45 (5%) of 853 babies were lost to follow-up (excluding deaths). In a clinical trial to determine the effect of prenatal vitamin A supplementation on perinatal transmission of HIV, the intervention was well received by pregnant women. The overall loss to follow-up during pregnancy was about 5% (enrollment started at 28 weeks gestation).

To minimize losses in previous studies, we introduced several approaches. We intend to follow the same follow-up strategies in this study. High follow-up rates have been facilitated by mapping of communities within Blantyre from which most of the study populations were drawn. The project nurses and clerks are experienced in conducting home visits to encourage women to return for their follow-up visits, and to ascertain the survival status of children. We are in the process of implementing a pilot Global Positioning System (GPS) study to assess if exact digital mapping can be introduced in our follow-up studies. We continuously evaluate causes of loss to follow-up in our studies. Certain factors such as child death and movement due to changes in family situations are inevitable. Lack of interest and refusals after being enrolled are potentially modifiable through adequate counseling, active tracing, adequate infrastructure development, and community participation. We have established Community Advisory Boards (CAB) to increase awareness and solicit community involvement.

D. 3. STUDY PRODUCT

D. 3. a. Active product. We will use metronidazole vaginal gel, 0.75% MetroGel-Vaginal (3M Pharmaceuticals) in this study. Based on a Clinical Monograph from the company (55), "MetroGel-Vaginal is an effective, well-tolerated local treatment for BV. Each gram of MetroGel-Vaginal contains 7.5 mg of metronidazole, 0.8 mg of methyparaben, 0.2 mg of propyl-paraben in an exclusive hydrogel vehicle consisting of purified water, propylene glycol, carbomer 934P, sodium hydroxide (to adjust pH), and edetate disodium. The gel is buffered to a normal pH of 4.0 and is free of mineral oil. Each applicatorful delivers about 5 grams of gel and contains approximately 37.5 mg of metronidazole in solution, thus, the pharmacologic activity of metronidazole is not limited by drug dissolution."

The product is used intravaginally either once (at bedtime) or twice a day. Both regimens have comparable efficacy (1). Clinical trials which compared intravaginal gel (5 gm BID for 5 days) vs oral metronidazole (500 mg BID for 7 days) have also shown comparable efficacy of about 84% after 11-17 days from start of therapy to 71% after 34-40 days from initiation of therapy (55). Despite its wide spread use, metronidazole is reported to be highly active and drug resistance is relatively rare (55). This is a desirable feature since we intend to use this drug intermittently for a longer period. Metro-Gel is reported to increase vaginal lactobacilli (55). We anticipate longer treatment to reduce recurrences and help to restore normal vaginal flora. It is estimated that 10%-15% of women who initially respond to therapy, relapse by one month after treatment, and 50%-70% of women develop recurrences after 3 months of initial therapy (1). Several causes have been postulated to account for these recurrences (1); for example, inability to reestablish normal *lactobacillus*-dominant protective flora, persistence of BV-causing bacteria and reinfection by a male partner.

D. 3. b. Placebo. The placebo will be manufactured to be similar to the active product but without the active ingredient. Identical consistency, color, smell and packaging will be maintained.

D.3. c. Provision of MetroGel and placebo. We have discussed this protocol with 3M Pharmaceuticals and the company has agreed to donate the treatment and the placebo for this study (see Appendix: letter from 3M Pharmaceuticals).

D. 3. d. Adverse experience (AE) reporting. We will record all AEs in designated forms and will grade them using standard methods. These AEs will be regularly reported to the PI.

D. 4. STATISTICAL CONSIDERATIONS

Primary endpoints. a) Incidence/Prevalence of BV at cross-sectional visits; b) Portion of pregnancies lost during the first 14 weeks of gestation; c) Incidence of adverse reactions to the product (portion of subjects having adverse reactions); and d) Rates of participation in study, adherence to treatment, and subsequent dropout.

Sample size and power. The following design specifications will apply: number of treatment groups: 2; follow-up period: approximately one year; hypotheses will be tested using a one-sided type 1 error of α=0.05. Unless stated otherwise, powers are obtained based on Fisher's exact test for comparisons of proportions in two groups using software developed by Dupont and Plummer (56).

We will recruit 1664 women of whom 832 will be HIV infected and 832 will be uninfected. In each group of women, 416 will be randomized to treatment arm and 416 to placebo arm. Based on previous experience in Malawi, losses due to dropouts and non-compliance will be less than 10% per year. Thus at least 375 women in each arm will return to each study visit during the first year.

Specific aim 1. Power estimates for specific aim 1 is shown in Tables 11. In previous studies from Malawi, cross sectional prevalence of BV among untreated women was 30% and that of any vaginal disturbance was 89% (24).

Table 11. Estimated power for specific aim 1 (comparing BV at each visit during the first study year assuming at least 375 evaluable study subjects in each group).

**Normative rate in untreated women**	**Reduction from intervention 33%**	**50%**
0.30	**.87**	~1.00
0.89	**~1.00**	~1.00

Thus there will be **0.87** and almost **1.00** power to detect 33% reductions in cross sectional visit prevalence of BV (normative rate 0.30 in untreated subjects) and any vaginal flora disturbance (normative rate .89 in untreated subjects).

Specific aim 2. Previous reports suggest that 20%-30% of women without treatment will lose their first post-index pregnancy (27). Table 12 presents powers to detect 33% and 50% reductions in the rates of early gestational loss of this first post index pregnancy based on 300 evaluable subjects in each group (we have assumed that 80% of these women will become pregnant again). There is virtual certainty to detect 50% reductions if pregnancy loss of 30% occurs, and >90% reduction if the rate of pregnancy loss is 20%. For a 33% reduction, there is about 80% power if the rate of loss is high (30%) and limited power if the rate of pregnancy loss is low (20%).

Table 12. Estimated power for specific aim 2 assuming 300 evaluable subjects in each group during the first year.

**Normative rate of pregnancy loss**	**Reduction from intervention 33%**	**50%**
0.20	**.54**	.91
0.30	**.78**	.99

Data management. Data entry and storage will be conducted on-site. The Johns Hopkins Project in Malawi has trained staff who have performed this activity for over a decade. Each record will be double entered into the computer and records compared. No direct subject identifiers (e.g., names, addresses, etc.) will be entered into data sets. The data will be stored on secure computers in Malawi and Johns Hopkins University. All original data collection forms will be kept in locked file cabinets. Data entry and management procedures are described further under Quality Control and Assurance (section D. 5). FoxPRO and SAS for windows are available for data management in Malawi. Computers used in the study are protected against computer virus attack by Norton antiviral software. All data sets are backed up onto tapes on a weekly basis both at Malawi and Johns Hopkins University(JHU). For this study we propose to manage the data locally in Malawi and perform further management and data analyses at JHU in Baltimore. The data will electronically be transferred to Baltimore.

Data analysis. Analyses will be done using an intention to treat approach. Separate analyses for HIV infected and uninfected women will be performed and the effect of the intervention on outcomes in these two groups will be compared. The effect of the intervention will be based on comparing incidence [2] of BV and portion of pregnancy losses between treated and untreated (control) subjects.

Multiple hypotheses will be tested in this study, requiring a diverse array of analytical methods

---

[2] Although we use the term incidence in the text, it is more accurate to define this measure as cross-sectional prevalence because the number of BV cases during a 3 month interval are a combination of new and continuing cases (due to incomplete cure and recurrences).

to compare the treatment and control arms. Descriptive comparisons will be made between the two study groups to identify potential confounding variables which could bias hypothesis testing through direct comparisons. When possible, as differences are noted, multivariate adjustment methods will be used to minimize the effect of confounders on testing the hypotheses. Continuous variables will be tested for skewness, kurtosis and outliers which could cause imprecision in standard asymptotic statistical methods. When such conditions occur, transformations such as logarithms, dichotomization or Winsorizing will be used, or nonparametric statistical methods will be implemented. For the statistical tests described below, both P-values and confidence intervals will be calculated for the parameters of interest.

Most outcomes of interest (e.g., presence of BV) are uncensored binary or categorical events. These include demographic and specific behavioral factors, etc. Contingency (n x 2) tables will be used to compare these events between the two study arms. Adjustment for covariates will be made with logistic or ordinal logistic regression. Comparison of binary events between two time-points within the same group will be done using methods that match on the same individual such as discordant pairs or conditional logistic regression.

In other outcomes of interest which are continuous comparison of mean values will be made using t-tests. Adjustments for covariates will be performed through linear regressions. Comparisons of continuous covariates for the same individuals in a group over different time periods will be made using paired z-tests and linear regressions on differences of values.

Some outcomes will be censored binary events. Standard survival analysis techniques such as Kaplan-Meier curves and Poisson regression will be used to directly compare outcome in both groups. These methods can also compare incidence rates within the same group over different time periods. Multivariate analogues that utilize covariates (proportional hazards models and Poisson regression) will adjust these comparisons for other covariates (57).

For some analyses we will pool repeated measurements from the same individual over multiple study visits. For example, occurrence of BV across all post-intervention study visits will be compared between study groups. For these analyses, generalized estimation equation models, which use an individual as cluster unit, will be fit to obtain robust covariance. For binary outcomes, a logit link will be used.

Data monitoring and reporting. This trial will be monitored by a Data and Safety Monitoring Board (DSMB). Formal interim analyses of efficacy of the intervention, based on the primary endpoints, will then be performed at approximately six month intervals during the projected study duration. Recommendations for early termination of positive or negative results will be guided by the symmetric group sequential O'Brien-Fleming Boundary (58). The O'Brien-Fleming design allows for early termination if extreme initial results are seen, while essentially allowing employment of the standard single stage one-sided 0.025-level test statistic at the final analysis if it is reached. This enables one to maintain the power of the single-stage design in the presence of interim monitoring without having to increase the maximum sample size. As has been the plan for other clinical trials conducted by our research team in Malawi, we will arrange for regular dissemination of information.

D. 5. QUALITY CONTROL AND ASSURANCE

Data management. Data entry programs are written on FoxPRO. The programs check out-of-range errors, skip pattern errors and verification errors before updating the master file. Further checking is done during data cleaning after data entry. Initially, checking of errors starts in the clinic where the forms are completed. A designated clerk reviews forms for incompleteness, inaccuracies and inconsistencies before the forms are sent for data entry. For quality control purposes, this clerk logs

the identification code on forms found with an error, interviewer's code, error type, form title and the specific question where the error was encountered. This monitoring is used to retrain interviewers who consistently make mistakes. At the data entry site in the office, forms are coded for missing data, not-applicable and open ended responses. During this process of coding, checking for errors continues. Written management guidelines are kept by the Data Managers in Malawi and at JHU in Baltimore.

Laboratory. The Johns Hopkins University-College of Medicine Research Project has a local laboratory capable of performing all conventional bacteriologic, hematologic and parasitologic tests. It also has a flowcytometry lab, and efforts are underway to establish a local PCR laboratory. We will use this laboratory in the proposed study. Adequate space is available for additional equipment and personnel. Strict safety and good clinical practice procedures will be followed in the laboratory. These regulations will be reinforced and supervised by the Field Director and the Project Coordinators on daily basis. Standard equipment will be used. Currently the Johns Hopkins Project in Blantyre performs routine maintenance through service contract with international agents. A quality assurance program has been developed for the local laboratory in Malawi. All tests are carried out according to written protocols. These protocols (e.g., HIV testing) follow the manufacturers recommendations and explicitly specify how to interpret the test results, and when to repeat a test if certain cut-offs points are not met. Selected specimens are tested in duplicate, at the Project laboratory in Blantyre and at the Johns Hopkins School of Hygiene and Public Health laboratories in Baltimore. Occasionally, an expatriate technician has visited the Project laboratory in Blantyre for purposes of re-training the technicians or introducing a new laboratory technique. Training sessions for technicians are regularly conducted with the start of any new study protocol, especially for unconventional tests.

D. 6. STRENGTHS AND LIMITATIONS OF THE PROPOSAL

A major strength of the proposed research is our familiarity with the site and available expertise and infrastructure in Malawi and at Johns Hopkins University in the U.S. We have extensively studied and documented bacterial vaginosis and STDs among Malawian women. We will use an appropriate design to determine the effect of the intervention - the intravaginal gel. This study will utilize the infra-structure and populations available for on-going screening activities to investigate an important maternal health problem. A potential limitation is inability of this study to adequately assess the effect of the intervention on reducing early pregnancy losses if these events are rare (e.g., 20% or less).

An aspect of this study which we will consider in our analysis is the potential reduction of HIV acquisition that can be associated with use of the active product. Our previous observational cohort studies among postnatal Malawian women have shown that BV is associated with increased acquisition of HIV. Therefore, this study could provide preliminary data to show if there is a trend of decrease in HIV seroconversion among women using the active gel compared with women using placebo. The study is not adequately powered to answer this question; nonetheless, detection of a trend can be the basis to justify a larger study.

This study could also provide preliminary data on reduction of *T. vaginalis* infections since metronidazole can treat this infection as well. This topical regimen (Metrogel), we realize, is unlikely to have a high efficacy against *T. vaginalis* compared with oral metronidazole since therapeutic levels in the urethra and perivaginal glands will not be achieved. However, reduction of genitourinary symptoms will be a desirable outcome.

D. 7. TIMELINE

Obtain approvals (~3 months)
x---------x

Enrollment postnatally (~6 months)
x--------------x

Follow-up (~18 months)
x---x

Analysis and study closure (~6 months)
x---------------x

x---------x--------------x--------------x---------------x
3/01 6/01 12/01 6/02 12/02
Calendar time

E. HUMAN SUBJECTS

The proposed study is a collaborative research between scientists from the U.S. and the Malawi College of Medicine. The main objective of the study is to determine the effect of using an intravaginal treatment gel on reducing genital tract infections and improving maternal health. The study will be conducted at several hospitals and health centers in and around Blantyre, Malawi (southeast Africa).

1. Involvement of human subjects:

About 1,664 women will be enrolled at 3 months postnatally. The inclusion criteria are: willingness to give a written informed consent to be tested for HIV and to be enrolled in the study; willingness to return for follow-up visits every 3 months for one year after enrollment; willingness to use the product as instructed; willingness to provide specimens for pregnancy and STD tests (including HIV for uninfected women); and resident of the study area. The exclusion criteria will include inability or unwillingness to follow any of the inclusion criteria.

2. Research material from human subjects:

Specimens collected will include blood from the mother at enrollment and at 3,6,9 and 12 months visits to test for syphilis and HIV (about 5 ml). Urine and vaginal fluid samples will be collected from the mother at each visit to detect pregnancy and sexually transmitted diseases (STDs). Data on risk factors will be collected at enrollment and at subsequent visits. The specimens and data are specifically obtained for research purposes and will be safely stored to ensure confidentiality.

3. Recruitment and consent procedures:

Study participants will be informed about the study when they present to the hospital for delivery or at the first postnatal visit. All participants will be fully counseled at enrollment about the study, STDs, and HIV testing (the meaning of a positive and a negative test will be explained), means of HIV transmission and the goals and procedures of the proposed study. The counseling will be carried out by trained counselors in the local language (Chichewa). All participants will sign (or thumb print, if illiterate) a written consent in the local language for HIV testing and to be enrolled in the study. Health care will not be compromised by refusal to participate.

4. Potential risks:

Physical risk. A physical risk might occur from phlebotomy (bruising). In order to minimize risk, blood will be collected by study staff trained in optimal techniques of blood drawing, infection control and health care worker protection. Only disposable needles and vacutainers will be used. Eligible participants will use an intravaginal gel or a gel without the active treatment agent. Participants will be informed that their chances of receiving the active gel is determined by chance (like flipping a coin); half of the women will receive the active gel and the other half will receive a placebo (the inactive gel). The gel is used intravaginally and is approved for treatment of bacterial vaginosis, the disease we are interested in evaluating in this study. The gel is used for five days only once every day at bedtime. No major risks have been reported when used in nonpregnant women. Minor complaints include yeast infection and vaginal irritation.

Psychological risk. These include risk of anxiety and depression during notification of HIV status. Trained counselors and study personnel will be available to provide appropriate counseling and clinical management. Referral to support and social services will be available.

Social risk. Social stigmatization based on knowledge of HIV status of the woman could occur. To avoid discrimination and to conceal the HIV status of the clients, counseling will be performed in private, and all participants (both HIV infected and uninfected) will attend the same clinic and undergo the same study procedures. Women will be free not to answer any question if they feel the information being requested is sensitive. Failure to respond will not impact provision of routine

medical care.

5. Confidentiality:

We will use ID numbers to keep history data and test results anonymous in Malawi. All information will be kept locked in safe cabinets in the project office in Blantyre. Only the field director and the data manager will have access to the forms. All test results will be kept confidential. Participants will be identified only by a code, and personal information will not be released.

6. Risks vs benefits:

We do not anticipate major risks. There could be beneficial effects as a result of this study. If the intervention is successful, a simple treatment regimen which can be used by women could be available to treat genital tract infections. It is likely that such a practical treatment approach could improve the health of mothers.

F. VERTEBRATE ANIMALS

Not applicable.

G. LITERATURE CITED

1. Hillier S, Holmes KK: Bacterial vaginosis. In: Sexually Transmitted Diseases, 3rd Edition. Edited by Homes KK, Sparling PF, Mardh P-A, Lemon SM, Stamm WE, Piot P, Wasserheit JN. New York: McGraw-Hill; 1999: 563-586.
2. Sewankambo N, Gray RH, Wawer MJ, Paxton l, McNairn D, Wabwire-Mangen F, Serwadda D, Li C, Kiwanuka N, Hillier SL, Rabe L, Gaydos CA, Quinn TC, Konde-Lule J. HIV-1 infection associated with abnormal vaginal flora morphology and bacterial vaginosis. Lancet 1997;350:546-550.
3. Govender L, Hoosen AA, Moodely J, Moodely P, Sturm AW. Bacterial vaginosis and associated infections in pregnancy. Int J Gynecol Obstet 1996;55:23-28.
4. Hillier SL. Diagnostic microbiology of bacterial vaginosis. Am J Obstet Gynecol 1993;169:455-459.
5. Eschenbach DA, Davick PR, William BL, Klebanoff SJ, Young-Smith K, Critchlow CM, Holmes KK. Prevalence of hydrogen peroxide-producing Lactobacillus species in normal women and women with bacterial vaginosis. J Clin Microbiol 1989;27:251-256.
6. Eschenbach DA. Bacterial Vaginosis. In: Hitchcok PJ, Mackay HT, Wasserheit JN, (Editors); Sexually Transmitted Diseases and Adverse Outcomes of Pregnancy. ASM Press, 1999; Washington, D.C., p.103.
7. Paavonen J. Physiology and ecology of the vagina. Scand J Infect Dis 1983;40(Suppl):31-35.
8. Hillier SL, Krohn MA, Nugent RP, Gibbs RS, the Vaginal Infections and Prematurity Study Group. Characteristics of three vaginal flora patterns assessed by Gram stain among pregnant women. Am J Obstet Gynecol 1992;166:938-944.
9. Holmes KK, Chen KCS, Lipinski CM, Eschenbach DA. Vaginal redox potential in bacterial vaginosis (nonspecific vaginitis). J Infect Dis 1985;152:379-382.
10. Hill JA, Anderson DJ. Human vaginal leukocytes and the effects of vaginal fluid on lymphocyte and macrophage defense functions. Am J Obstet Gynecol 1992;166:720-726.
11. Voller B, Anderson DJ. Heterosexual transmission of HIV (Letter) JAMA 1992;267:1917-1918.
12. Hillier SL, Krohn MA, Rabe L, Klebanoff SJ, Eschenbach DA. The normal vaginal flora, producing lactobacilli, and bacterial vaginosis in pregnant women. Clin Infect Dis 1993;16(Suppl):S273-81.
13. Barbone, FJ, Austin H, Loew WC, Alexander WJ. A follow-up study of methods of contraception, candidiasis, and bacterial vaginosis. Am J Obstet Gynocol 1990;163:510-514.
14. Colli H, Landoni M, Parazzini F, and participants. Treatment of male partners and recurrence of bacterial vaginosis: a randomised trial. Gentourin Med 1997;73:267-270.
15. Moi H, Erkkola R, Jerve F, Nelleman G, Bymose B, Alaksen K, Tornqvist E. Should male consorts of women with bacterial vaginosis be treated? Genitourin Med 1989;65:263-268.
16. Hillier SL, Nugent RP, Eschenbach DA, Krohn MA, Gibbs RS, Martin DH, Cotch MF, Edelman R, Pastorek JG, Rao AV, McNellis D, Regan JA, Carey JC, Klebanoff MA. Association between bacterial vaginosis and preterm delivery of a low-birth-weight infant. N Engl J Med 1995;333:1737-42.
17. Hillier SL, Martius J, Krohn MA, Krohn M, Kiviat N, Holmes KK, Eschnbach DA. A case controlled study of chorioamniotic infection and histologic chorioamnionitis in prematurity. N Engl J Med 1988;319:972-988.
18. Gravett MG, Nelson HP, DeRouen T, Critchlow C, Eschenbach DA, Homes KK. Independent associations of bacterial vaginosis and chlamydia trachomatis infection with adverse

pregnancy outcome. JAMA 1986;256:1899-1903.
19. Gravett MG, Hummel D, Eschenbach DA, Homes KK. Preterm labor associated with subclinical amniotic fluid infection and with bacterial vaginosis. Obstet Gynecol 1986;67:229-237.
20. Minkoff H, Grunebaum A, Feldman J, Cummings M, McCormack W. Relationship of vaginal pH and Papanicolaou smear results to vaginal flora and pregnancy outcome. Int J Gynecol Obstet 1987;25:17-23.
21 Eschenbach DA, Hillier S, Critchlow C, Stevens C, DeRouen T, Holmes KK. Diagnosis and clinical manifestations of bacterial vaginosis. Am J Obstet Gynecol 1988;158:819-28.
22 Eschenbach DA, Bacterial vaginosis: emphasis on upper gential tract complications. Obstet Gynecol Clin NA 1989;16:593-610.
23 Martin Jr HL, Richardson BA, Nyange PM, Lavreys L, Hillier SL, Chohan B, Mandaliya K, Ndinya-Achola JO, Bwayo J. Kriess J. Vaginal Lactobacilli, Microbial Flora, and Risk of Human Immunodeficiency Virus Type 1 and Sexually Transmitted Disease Acquisition. J Infect Dis 1999;180:1863-1868.
24. Taha TE, Hoover DR, Dallabetta GA, Kumwenda NI, Mtimavalye LAR, Yang LP, Liomba GN, Broadhead RL, Chiphangwi JD, Miotti PG. Bacterial vaginosis and disturbances of vaginal flora: association with increased acquisition of HIV. AIDS 1998;12:1699-1706.
25 Cohen CR, Duerr A, Pruithithada N, Rugpao S, Hillier S, Garcia P, Nelson K. Bacterial vaginosis and HIV seroprevalence among female commercial sex workers in Chiang Mai, Thailand. AIDS 1995;9:1093-1097.
26. Taha TE, Kumwenda N, Liomba H, Chiphangwi J, Dalabetta G, Hoover D, Miotti P. Heterosexual and perinatal transmission of HIV-1: associations with bacterial vaginosis (BV). World AIDS Conference, Geneva, Switzerland, July 1998: (Abstract 527).
27. Wilcox AJ, Weinberg CR, O'Connor JF, Baird DD, Schlatterer JP, Canfield RE, Armstrong EG, Nisula BC. Incidence of early loss of pregnancy. N Engl J Med 1988;319:189-194.
28. Ralph SG, Rutherford AJ, Wilson JD. Influence of bacterial vaginosis on conception and miscarriage in the first trimester: cohort study. BMJ 1999;319:220-223.
29. Hay PE, Lamont RF, Taylor-Robinson D, Morgan DJ, Ison C, Pearson J. Abnormal bacterial colonisation of the genital tract and subsequent preterm delivery and late miscarriage. BMJ 1994;308:295-298.
30. McGregor JA, French JI, Jones W, Milligan K, McKinney PJ, Patterson E, Parker R. Bacterial vaginosis is associated with prematurity and vaginal fluid mucinase and sialidase: Results of a controlled trial of topical clindamycin cream. Am J Obstet Gynecol 1994;170:1048-1060.
31. Gray RH, Wawer MJ, Serwadda D, Sewankambo N, Li C, Wabwire-Mangen F, Paxton L, Kiwanuka N, Kigozi G, Konde-Lule J, Quinn TC, Gaydos CA, McNaim D. Population-based study of fertility in women with HIV-1infection in Uganda. Lancet 1998;351:98-103.
32 CDC. 1998 Guidelines for treatment of sexually transmitted diseases. MMWR 1998;47 No. RR-1:7-74.
33 Joesoef MR, Hillier SL, Wiknjosastro G, Sumampouw H, Linnan M, Norojono W, Idajadi A, Utomo B. Intravaginal clindamycin treatment for bacterial vaginosis: Effects on preterm delivery and low birth weight. Am J Obstet Gynecol 1995;173:1527-1531.
34. Taha TE, Gray RH, Kumwenda NI, Hoover DR, Mtimavalye LAR, Liomba GN, Chiphangwi JD, Dallabetta GA, Miotti PG. HIV infection and disturbances of vaginal flora during pregnancy. J Acquir Immune Defic Syn Hum Retrovirol 1999;20:52-59.

35. Taha TE, Dallabetta GA, Hoover DR, Chiphangwi JD, Mtimavalye LAR, Liomba G, Kumwenda NI, Miotti PG. Trends of HIV-1 and sexually transmitted diseases among pregnant and postpartum women in urban Malawi. AIDS 1998; 12:197-203.
36. Ulin PR. African women and AIDS: negotiating behavioral change. Soc Sci Med 1992;34:63-73.
37. Dallabetta GA, Miotti PG, Chiphangwi JD, Liomba G, Saah AJ. Traditional vaginal agents: prevalence of use and associations with HIV-1 infection in Malawian women. AIDS 1995;9:293-297.
38. Taha TET, Canner JK, Chiphangwi JD, Dallabetta GA, Yang L-P, Mtimavalye LAR, Miotti PG. Reported condom use is not associated with incidence of sexually transmitted diseases in Malawi. AIDS 1996;10:207-212.
39. Taha TE, Dallabetta GA, Canner JK, Chiphangwi JD, Liomba G, Hoover DR, Miotti PG. The effect of human immunodeficiency virus infection on birthweight, and infant and child mortality in urban Malawi. Int J Epidemiol 1995;24:1022-1029.
40. Miotti PG, Dallabetta GA, Chiphangwi JD, Liomba G, Saah AJ. A Retrospective study of childhood mortality and spontaneous abortion in HIV-1 infected women in urban Malawi. Int J Epi 1992;21:792-799.
41. Taha TE, Kumwenda NI, Broadhead RL, Hoover DR, Graham SM, Van der Hoeven L, Markakis D, Liomba GN, Chiphangwi JD, Miotti PG. Pediatr Inf Dis J 1999;18:689-694.
42. Taha TE, Kumwenda NI, Hoover DR, Biggar RJ, Broadhead RL, Cassol S, Van der Hoeven L, Markakis D, Liomba GN, Chiphangwi JD, Miotti PG. Association of HIV-1 load and CD4 lyphocyte count with mortality among untreated African children over one year of age. AIDS 2000;14:453-459.
43. CDC.1994 Revised classification systems for human immunodeficiency virus infection in children less than 13 years of age. MMWR 1994; 43(N0. RR-12):1-19.
44. Taha TE, Graham SM, Kumwenda NI, Broadhead RL, Hoover DR, Biggar RJ, Markakis D, Van der Hoeven L, Liomba GN, Chiphangwi JD, Miotti PG. Morbidity among HIV-1 infected and uninfected African children. Pediatr *(in press)*.
45. Taha TET, Miotti P, Liomba G, Dallabetta G, Chiphangwi J. HIV, maternal death and child survival in Africa. AIDS 1996;10:111-112.
46. Biggar RJ, Miotti PG, Taha TE, Mtimavalye L, Broadhead R, Justesen A, Yellin F, Liomba G, Miley W, Waters D, Chiphangwi JD, Goedert JJ. Perinatal intervention trial in Africa: effect of a birth canal cleansing intervention to prevent HIV transmission. Lancet 1996;347:1647-1650.
47. Semba RD, Kumwenda N, Hoover DR, Taha TE, Quinn TC, Mtimavalye L, Biggar RJ, Broadhead R, Miotti PG, Skoll L, Hoeven LVD, Chiphangwi JD. Human Immunodeficiency viral load in breastmilk, mastitis, and mother-to-child transmission of human immunodeficiency virus type 1. J Infect Dis 1999; 180:93-98.
48. Miotti PG, Taha TE, Kumwenda NI, Broadhead R, Mtimavalye LAR, Hoeven LVD, Chiphangwi JD, Liomba G, Biggar RJ. HIV transmission from breastfeeding: a study from Malawi. JAMA 1999; 282:744-749.
49. Semba RD, Miotti PG, Chiphangwi JD, Canner J, Saah AJ, Dallabetta GA, Hoover DR. Maternal vitamin A deficiency and mother-to-infant transmission of HIV-1. Lancet 1994; 343:1594-1597.
50. Taha TE, Biggar RJ, Broadhead RL, Mtimabalye LAR, Justesen AB, Liomba GN, Chiphangwi JD, Miotti PG. Effect of cleasing the birth canal with antiseptic solution on maternal and newborn morbidity and mortality in Malawi: clinical trial. BMJ 1997;315:216-220.

51 Guay LA, Musoke P, Fleming T, Bagenda D, Allen M, Nakabiito C, Sherman J, Bakaki P, Ducar C, Deseyve M, Emel L, Marochnick M, Fowler MG, Mofenson L, Miotti P, Dransfield K, Bray D, Mmiro F, Jackson JB. Intrapartum and neonatal single-dose nevirapine compared with zidovudine for prevention of mother-to-child transmission of HIV-1 in Kampala, Uganda: HIVNET 012 randomized trial. Lancet 1999;354:795-802.

52. van de Wijgert J, Fullem A, Kelly C, Mehendale S, Kumwenda N, Rugpao S, Joshi S, Taha T, Nelson K, Padian N. Phase I trial of the topical microbicide BufferGel: Safety results from four international sites. Microbicide 2000 Workshop, Washington DC, March 13-16, 2000.

53. Nugent RP, Krohn MA, Hillier SL. Reliability of diagnosing bacterial vaginosis is improved by a standardized method of grain stain interpretation. J Clin Microbiol;1991;29:297-301.

54. Amsel R, Totten PA, Spiegel CA, Chen KCS, Eshenbach DA, Holmes KK. Nonspecific vaginitis - diagnostic criteria and microbial and epidemiologic associations. Am J Med 1983;74:14-22.

55. 3M Pharmaceuticals. MetroGel-Vaginal. Effective intravaginal therapy for bacterial vaginosis. A Clinical Monograph, 1997.

56. Dupont WD, Plummer WD. Power and sample size calculations: A Review and computer program. Controlled Clin Trials 1990;11:116-128.

57. Brown CL, Green SB. Additional power computations for designing comparative Poisson trials Am J Epidemiol 1982; 115:752-8

58. O'Brien PC, Fleming TR. A multiple testing procedure for clinical trials. Biometrics 1979;35:549-556.

INTRAVAGINAL TREATMENT OF DISTURBANCES OF VAGINAL FLORA AMONG HIV INFECTED AND UNINFECTED WOMEN IN MALAWI

THE METRO STUDY

Blantyre, Malawi

Manual of Operations (MOP)

Principal Investigators
Taha E. Taha MD PhD, Department of Epidemiology, School of Hygiene and Public Health, Johns Hopkins University, Baltimore, MD
Newton I. Kumwenda PhD, Johns Hopkins University-Malawi College of Medicine Research Project, Blantyre, Malawi

Co-Investigators
George Liomba MBBS FRCPath, Pathologist
Valentino Lema, MBChB, MMed O&G, Obstetrician & Gynecologist
Joyce Munthali, MBChB, MMed O&G, Obstetrician & Gynecologist
George Kafulafula MBBS MMed O&G, Obstetrician & Gynecologist
Eyob Tadesse MD PhD, Obstetrician

College of Medicine, University of Malawi

Protocol Statistician
Donald Hoover PhD, Rutgers University, NJ, USA

Study Coordinator
Chiwawa Nkoma MSc, Johns Hopkins University Research Project, Blantyre Malawi

Last Revision: February 10, 2003

TABLE OF CONTENTS

1.0 Introduction 3

2.0 General rules for project work 5

3.0 Study design 6

4.0 Procedure for HIV screening 12

5.0 Procedures for obtaining informed consent and enrollment 14

Procedures for physical exam 16

Procedures for collection, testing & storage of blood samples 18

Procedures for collection & testing of vaginal and urine samples 20

General guidelines for shipping of samples 21

10.0 Study forms and documentation procedures 23

Study treatment 26

Statistical considerations 28

Addendums* 32

APPENDIX 1. HIV Testing Consent Forms (NVAZ, Chorio and 016A studies)

APPENDIX 2. METRO Study Consent Forms: HIV Screening, Enrollment, Male Focus Group Discussion, Outline of Focus Group Discussions (both men and women group discussions), & Medical Release Consent Forms

APPENDIX 3. Study Forms

APPENDIX 4. Assessment of Adverse Experience

** Addendums include revisions to standard operating procedures that have been added to this manual during the course of the study.*

1.0. INTRODUCTION

1.1 The Manual of Operations

This manual introduces the staff of the Johns Hopkins Project, College of Medicine, and others to the activities and procedures to be carried out in the study clinic and community health centers relating to the METRO study. This manual explains the purpose and organization of the study and describes in detail the procedures and forms that must be known for the different sites involved in the study. This manual is a required reading for all study personnel. All nurses involved in the study must undergo certification before involvement in the study, and a working knowledge of this manual is necessary.

1.2 Purpose of the Study

Vaginal flora disturbances are common among women of reproductive age in both developing and developed countries. Our previous studies in Malawi showed that only about 11% of childbearing women had a normal vaginal flora and 89% had a vaginal disturbance which ranged from mild to severe. Several adverse reproductive outcomes are associated with "bacterial vaginosis (BV)", a severe disturbance of vaginal flora. Therefore, identifying a simple treatment regimen for these vaginal disturbances is highly desirable for Malawian women and others in similar settings. We hypothesize that in areas where BV is common, an antibiotic treatment regimen will restore normal flora, reduce vaginal disturbances and their recurrences. Such a measure is simple and woman-controlled. This intervention has the potential to reduce adverse reproductive outcomes and improve maternal health.

1.3 Organization of the Study

The study is made up of health professionals, scientists, administrators, supervisors, field staff, and computing and support staff. The main study locations are the project clinic at Queen Elizabeth Central Hospital (QECH) and the health centers around Blantyre district, Blantyre, Malawi. The main study laboratory and specimen repository is the Johns Hopkins Project laboratory at QECH.

1.4 Brief Summary of Activities

All of the study activities are carried out at QECH, the health centers and other participating health institutions. Participants will be recruited postnatally into the proposed study from women who had already been screened for HIV either antenatally in the chorioamnionitis treatment study or at delivery in the NVAZ study. HIV infected women and HIV uninfected women who were screened in these trials, but were not eligible for enrollment, will also be recruited into the current Metro trial. Study participants will be seen postnatally from enrollment at 3-months after delivery, and at quarterly visits thereafter for a duration of 12 months (or 15 months from time of delivery).

1.5 Advantages of the proposed enrollment scheme

Recruitment of women from the on-going NVAZ and chorioamnionitis perinatal trial populations has several advantages:

- Participating women will benefit from the trial (we will screen for genital infections and provide treatment if indicated);
- Is cost-effective because the existing infra-structure of clinic personnel, counseling and testing, and the same child/mother follow-up arrangements will be utilized;
- Involvement of women in on-going research for the baby could increase rates of retention in these studies because women will become active participants;
- Enrolling both HIV infected and un-infected women in these clinics will maintain confidentiality of participants and eliminate potential discrimination based on HIV status of participants.

2.0. GENERAL RULES FOR PROJECT WORK

2.1 Work honestly.

This is the most basic rule for doing work in the study. Only honest work can let the project properly evaluate the effectiveness of antibiotic treatment in reducing vaginal disturbances among HIV infected and uninfected women. This is such an important rule that it will also serve as a basis for staying employed by the project. Information must always be collected honestly. Honest mistakes can be tolerated. Dishonesty, or making up information on forms, can never be tolerated. Therefore, a worker who is found to be dishonest in his or her work will immediately be terminated by the project.

2.1.1. Follow the instructions.

In this study, you will dispense the study treatments as instructed. All information about study clients is strictly confidential. It is unethical to deviate from these instructions; this will result in termination of your work with the Project. The study coordinators, the field director and the investigators will regularly monitor and reinforce these instructions.

2.2 If you are not certain, please ask.

You are encouraged to ask and understand study procedures. Always inquire from your superiors if you are in doubt. Do not feel obliged to take a decision if you can not understand a specific instruction or a study procedure as explained in this manual.

2.3 Be courteous at all times.

Each staff member represents the project whether on or off the job, and being courteous at all times helps to maintain vital hospital and community support for the project. The desire of the patients to enroll and continue follow-up in the project depends a lot upon the attitude of the staff members.

2.4 Be prepared.

Always check that you have the correct forms, the correct number of forms, and the supplies that you need before you begin working with the patients in the study. In the same way, always check that you have all of your materials packed with you before you leave, especially those making any visits to the community or community health centers.

2.5 Be on time.

Arrive at the clinic at the time you are expected. Timeliness reflects a proper attitude and commitment towards the job. Excessive tardiness and absenteeism are also grounds for termination of employment in this project.

2.6 Be careful.

Do your work with care and attention to detail. You will often be doing the same tasks from month to month. Approach each day with a freshness that comes from knowing that you are working with new patients each day even though the procedures may be the same. If the information is collected carefully, the project will succeed in its goals. If information is collected

sloppily, the project will fail, and with it the hopes that this information could be used to help save lives in the future.

3.0. STUDY DESIGN

3.1 Design summary

3.1.1 Objectives:

- To determine the effect of postnatal intravaginal metronidazole gel treatment (once daily for 5 consecutive days every three months) on the incidence of BV among HIV infected and uninfected women.
- To determine the effect of mass treatment of BV on rates of conception and early pregnancy loss among HIV infected and uninfected women of reproductive age.
- To assess the clinical and behavioral factors which facilitate or impede the long-term use of metronidazole vaginal gel in a community where use of traditional vaginal agents is common.

3.1.2 Type of study:

This is a randomized, double masked, placebo-controlled clinical trial. The study will have two arms: randomly assigned women will receive either an intravaginal gel with the active product or an intravaginal gel without the product (placebo). A mass treatment approach is used because the overall prevalence of disturbances of vaginal flora is high among Malawian women of childbearing age (89%). See Figure 1 below for study details.

Figure 1. Recruitment and follow-up.

INDEX → → → → → → → → → →
DELIVERY ——x——x——x——x——x——x
1.5 3 6 9 12 15 Months
Enrollment Follow-up Visits

3.1.3 Intervention:

Women (both HIV positive and negative) will be randomized at the three months postnatal visit and issued the intervention (either metronidazole gel or placebo). We will use metronidazole vaginal gel, 0.75% MetroGel-Vaginal (3M Pharmaceuticals) in this study. The product is used intravaginally once a day at bedtime for five consecutive nights. The placebo is similar to the active product but without the active ingredient.

3.1.4 Study population:

The study will be conducted at QECH and the health centers around the city of Blantyre. Eligible women are those who are screened in the on-going NVAZ and Chorio studies.

3.1.5 Inclusion criteria

- Willingness and ability to provide informed consent
- Willingness to take treatment (active product or placebo) as instructed
- Willingness to return for follow up visits
- Willingness to provide specimens for pregnancy and STD tests (including HIV for seronegative women)
- Resident of the study area.

3.1.6 Exclusion criteria

- Inability to provide informed consent
- Refusal of HIV testing
- Woman's desire to move from the study area
- Pregnant at enrollment
- Participation in other microbicide studies.

3.2. Data collection and visit schedules

Visit Number / Study Procedure	Screening	V 1	V1.9	V 2	V2.9	V3	V3.9	V4	V4.9	V5
**Obtain informed consent**	x	x								
**Enrol**		x								
**Randomize**		x								
**Issue product**		x		x		x		x		
**Assess compliance***			x		x		x		x	
**Questionnaires:**										
- Baseline		x								
- Follow-up			x**	x	x**	x	x**	x	x**	x
**Examination & anthropometry:**										
- Physical including pelvic exam		x		x		x		x		x
- Weight		x		x		x		x		x
- Height		x								
**Laboratory procedures:**										
- HIV testing***/Archiving	x	x		x		x		x		x
- Syphilis serology		x		x		x		x		x
- Vaginal wet mount:		x		x		x		x		x
- Vaginal pH		x	xx	x	xx	x	xx	x	xx	x
- Clue cells		x	xx	x	xx	x	xx	x	xx	x
- Whiff test		x	xx	x	xx	x	xx	x	xx	x
- T. vaginalis		x		x		x		x		x
- Candida (hyphae & buds)		x		x		x		x		x
- Archive cervico-vaginal swab		x		x		x		x		x
- Vaginal smear (for Gram stain)		x	xx	x	xx	x	xx	x	xx	x
- Urine pregnancy testing		x	xx	x	xx	x	xx	x	xx	x
- Hemoglobin level		x		x				x		
**Male/Female FGD¶**				x						x

Assessment of compliance will include returning and checking used product for tube ID label and amount used at each visit.

** Complete Acceptability History and Product Monitoring and Genital History Follow-up Forms.

*** HIV negative women only; at enrollment all women must have a confirmed HIV result either from previous studies or by testing before enrollment if a participant is a new client.

¶ Focus group interviews with 4-6 females and their male partners.

3.2.1 Visit numbering

The METRO study will use a sequential visit numbering starting from V1 as shown below.

Please note: The first METRO study visit starts postnatally at 3 months; this will represent V 1. Each quarterly visit is followed by a test of cure visit one month after using the product; these one month follow-up visits are coded as 1.9, 2.9, 3.9, 4.9 (NO V 5.9 because no product is used at V5). The purpose of test of cure visits is to collect vaginal samples to determine if there has been changes in vaginal flora following use of the study product. We will also complete Acceptability and Product Monitoring forms at these visits.

The METRO study visit numbers are as follows:

V 1: Visit at 3 months postnatal; followed by a test of cure visit after one month (i.e., at 4 months postnatal) - ***this visit is coded V1.9***.

V 2: Visit at 6 months postnatal; followed by a test of cure visit after one month (i.e., at 7 months postnatal) - ***this visit is coded V2.9***

V 3: Visit at 9 months postnatal; followed by a test of cure visit after one month (i.e., at 10 months postnatal) - ***this visit is coded V3.9.***

V 4: Visit at 12 months postnatal; followed by a test of cure visit after one month (i.e., at 13 months postnatal) - ***this visit is coded V4.9.***

V 5: Visit at 15 months postnatal. This is the last follow-up visit (remember that the test of cure visit should occur one month after V4 when the last tube is issued).

3.2.2 Two weeks rule for scheduled visits

A window of 2 weeks before or after the scheduled visit date will be considered for V2, V3, V4 and V5. For example, a woman coming to the clinic for a follow-up visit two weeks earlier (or later) than the expected scheduled date at 9 months postnatal will be assigned V3. This rule applies for both regular quarterly visits (V1, V2, V3, V4, V5) and the test of cure visits (V1.9, V2.9, V3.9, V4.9).

3.2.3 Women seen after the window of 2 weeks (Missed Visits)

For women seen after the window of 2 weeks, complete a missed visit form and assign the missed visit # on the form. **However, the test of cure visit (1.9, 2.9, 3.9 and 4.9) cannot be missed.** If a woman has missed her test of cure visit (taking into account ±2 weeks of the scheduled date), a missed visit form with all other forms to be completed at a regular rtest of cure visit should be completed,

and vaginal samples should also be collected. Specifically, the following should be done: A) complete Missed Visit Form, B) complete Acceptability History Form, C) Complete Product Monitoring and Genital History Form, C) Collect Vaginal mount and smear (for Gram stain), and do urine pregnancy testing.

Important Note: If the client presents at a time when she is due for her regular visit after missing the test of cure visit, all the above procedures should be done (from A to C) and the visit # should be that of the missed visit (e.g., 2.9 or 3.9). In addition, complete regular visit forms and collect other specimens such as STD samples and blood - on these forms indicate the regular visit number (not the missed visit number).

Example of a missed quarterly visit and of a missed test of cure visit:
1. Client comes for V1 on January 1 and V1.9 on February 1; then skips V2 on April 1 and does not return until May 15. This client should be re-scheduled for V3 on July 1. A missed visit 2 form should be completed. **Because this client came for the test of cure visit, we will only document that V2 was missed (remember that by missing V2 this client did not receive product and therefore we will not complete a product monitoring or acceptability/genital history forms, and will not collect samples). We are re-scheduling this client to a regular visit on July 1 to avoid irregularities with scheduling (i.e., if we consider this as a regular visit, all future visits will be changed).**

2. Client above skips the test of cure visit on February 1 and returns for her V2 on April 1. **As we explained above a test of cure cannot be missed; therefore complete the Acceptability Form, Product Monitoring/Genital History Form, and collect vaginal samples. These forms should be coded as V1.9. In addition, complete all other follow-up study forms and collect all other laboratory samples; code these forms as V2.**

3.2.4 Unscheduled visits

Unscheduled visits will be identified by visit date in addition to the usual identifiers such as ID number.

3.3 Clinic ID code

Each participating clinic including QECH will have an identification code. This code will appear on all forms and local laboratory specimens. In the METRO study the clinic codes are as follows:

QECH	1
Limbe	2
Cholomoni	3
Zingwangwa	6
Ndirande	7

3.4 METRO Study IDs

The METRO study will have a five digit numerical ID. The 5 digits represent the following: the first digit from the left represents the clinic ID (e.g., 1 for QECH, 2 for Limbe, etc.); the second digit represents HIV status of the woman (1 for HIV negative and 2 for HIV positive); and the third to the fifth digits represent random ID numbers for clients in each clinic ranging from 001 to 500. The numerical IDs will be preceded by 2 characters reading "MG" (e.g., MG11001). For simplicity, the study forms will show only the five numerical ID numbers, but each form will be clearly marked "METRO Study". IDs will be entered in the appropriate boxes (ID boxes are shown on each study form) as follows: e.g., 11001 - not 11,001. The original NVAZ or Chorio study IDs will be entered also on a separate box on the first page of enrollment study form. ***Please note: DO NOT MIX NVAZ OR CHORIO STUDY IDs WITH THE METRO STUDY IDs. EACH STUDY HAS A UNIQUE ID SERIES. BE CAREFUL!!!***

3.5. IMPORTANT: ID code and labelling of treatment assignment

This is a masked (blinded) clinical trial - neither you, the investigators or the patient would know who is receiving the Metornidazole or placebo gel. The tube which contains the gel will have the same ID number as assigned on the forms. These IDs are assigned randomly. Each treatment tube will have the following labels: ID # (e.g., MG11001); The METRO Study, and a visit number (e.g., V1, V2, V3, etc.) to show the visit number at which the tube was issued (remember: each woman will receive 4 tubes during the 12 months METRO study period). See example below showing the label which appears on each tube.

**MG11001**<br>**THE METRO STUDY**<br>**V1**

4.0. PROCEDURE FOR HIV SCREENING

Women to be enrolled in the METRO study would have been counselled and screened for HIV in the NVAZ or Chorio studies, respectively, at time of delivery or antenatally. The HIV screening described here is the same as the one used in the NVAZ or Chorio studies.

4.1. HIV screening

4.1.1 Purpose

- to give introductory information about the project to the mothers
- to counsel women for HIV testing
- to accurately identify women who agree to be HIV tested
- to allow accurate identification of women who are willing to be enrolled in the study
- to obtain written, informed consent for HIV screening (as mentioned previously, the same NVAZ screening consent form is used).

4.1.2 Forms

Two HIV screening consent forms are used in the NVAZ study at time of delivery, and one HIV screening consent form is used antenatally in the Chorio study (See Appendix 1 for a copy of the NVAZ and Chorio screening consent forms).

When/where

When women present for delivery or attend first antenatal visit at QECH or health centers around Blantyre.

By whom

Project nurses.

Eligibility

- resident in Blantyre or surrounding Districts (a study area)
- signed consent form for HIV testing
- willing to receive HIV counselling and learn results of HIV testing
- not participating in other microbicide studies.

4.2. HIV screening test

4.2.1. By whom

- The clinic nurses will collect either maternal blood or infant cord blood for HIV screening.
- Project nurses will perform rapid HIV testing. The Johns Hopkins Project (JHP) laboratory technicians will carry-out confirmatory HIV testing (see Section 7.0).

4.2.2. Supplies needed

- Lancets
- Vacutainers tubes
- Gauze pads
- Rapid HIV test kit.

4.2.3 Description of the procedure

For women who present antenatally or early at time of delivery, a finger prick maternal blood will be collected for rapid HIV testing. For women who arrive late at time of delivery (when there is no adequate time to conduct HIV counseling and testing before delivery) the project nurses will collect infant cord blood (from the placental side of the cord) in a red top vacutainer. This blood will be stored in a locked cabinet until informed consent is obtained from the woman. If the woman consents to be in the study, the infant's cord blood will be tested for HIV using a rapid test (see Section 7.0). If the woman does not consent, the stored blood sample will be properly and safely discarded.

4.2.4 Post-test counselling

Women will be post-test counselled as soon as the results of rapid HIV test are available. The women will be recounselled when the result of the confirmatory HIV test is available.

5.0. PROCEDURES FOR OBTAINING INFORMED CONSENT AND ENROLLMENT IN THE METRO STUDY

As indicated earlier, woman currently being screened for the NVAZ or Chorio studies will be eligible for enrollment in the METRO study. Although no formal consent form will be signed until 3 months after delivery (i.e., at V 3), potential participants will have the opportunity to hearing about the METRO study on several occasions during the general health talks provided antenatally and postnatally for all women attending any of the study clinics. JHP nurses will conduct health talks antenatally and postnatally and provide a brief description about the METRO study in Chichewa (local language). Interested women will be advised that they will be formally counselled to enrol at 3 months after delivery.

5.1 Purpose

To explain the project to women and to offer them the opportunity to participate in the METRO study if they understand the protocol and desire to participate.

How

All women will receive a health talk on issues related to mother and child health including contraception, breastfeeding and HIV transmission. This will be followed by an individual confidential counselling to know about the study details including visit schedules and procedures involved, and to confirm willingness to enroll in the METRO study. See Appendix 2: copy of METRO enrollment consent form.

5.3 By whom

Project nurses trained and certified in the principles of counselling and obtaining informed consent.

5.4 To whom

To women who fulfil the eligibility criteria to be enrolled in the study.

5.5 Informed consent procedure

- Explain the purpose of the study
- Explain the procedures and visits which are involved in the study
- Explain any risks and benefits involved with the study
- Answer the woman's questions and address any concerns
- Any participant who decides to enroll in the study must provide a written informed consent or thumb-printed consent
- A copy of the consent form is provided to all participants.

Storage of consent forms

- Consent forms are taken to the project office at the end of each day
- Signed consent forms should be filed in chronological order, by ID number in the project office
- Consent forms should be kept in a locked file

The Field Director (or designee) will make periodic checks of the consent forms and initial checked forms.
Any missing consent forms are considered a violation of ethical guidelines and the responsible individuals will be subject to disciplinary action.

5.7 Enrollment procedures

Each woman enrolling in the METRO study is systematically assigned to next available ID. For example, if the first HIV negative woman is assigned ID 11001, the next HIV negative woman should be assigned ID 11002. Similarly, if the first HIV positive woman is assigned ID 12001, the next HIV positive woman should be assigned ID 12002.

Each clinic will receive two separate lists of IDs; one list of IDs for randomization of women who are HIV negative, and another list of IDs for randomization of HIV positive women. See listing below for each clinic::

Clinic	Code	ID Range HIV -ve	ID Range HIV+ve
QECH	1	11001-11500	12001-12500
Limbe	2	21001-21500	22001-22500
Cholomoni	3	31001-31500	32001-32500
Zingwangwa	6	61001-61500	62100-62500
Ndirande	7	71001-71500	72001-72500

Within these ID ranges assigned to each clinic, women are randomized to receive either the active product or placebo.

6.0 PROCEDURES FOR PHYSICAL EXAM

6. A general physical clinical examination and a pelvic examination will be performed.

6.2 Purpose
To document baseline physical condition of the study participant and to detect any abnormal clinical conditions during the progress of the study. A systematic clinical evaluation of the participant will include examination of the head and neck (including oral exam), upper limbs, thorax, abdomen and groin, and lower limbs. The examination will also include external genitalia, a speculum-aided vaginal inspection, and bimanual vaginal exam.

6.3 When
At enrollment and each subsequent visit

6.4 Where
At the participating clinics.

6.5 By whom
Project nurses trained and certified in study procedures.

6.6 To whom
Women who have consented to be enrolled in the METRO study.

6.7 Supplies needed
- Thermometer
- Sphygnomanometer
- Speculum
- Adult weight scale
- Measuring tape
- Adjustable good light source.

6.8 Description of procedures
- Women will be informed of the procedure to be undertaken at each exam session.
- Weight and height are obtained in a standing position. The weighing scale will be regularly checked and recalibrated as necessary.
- Participant will rest comfortably on a standard examination couch.
- Temperature and blood pressure are measured.
- A routine general physical exam is performed and followed by a pelvic exam.
- The following steps are followed in a pelvic exam (record all findings adequately on appropriate forms):

- Palpate inguinal lymph nodes for enlargement and tenderness
- Visually inspect vulva and perineum for ulcers, rashes, vesicles, warts, or other lesions. Examine the genitalia and surrounding skin for evidence of scratching due to itching
  Inspect vaginal introitus for discharge and odor; note redness, itching marks, etc.
- Insert a speculum lubricated with water into the vagina
  Inspect visually the inside of the vagina and cervix for discharge (describe color, odor, source, quantity), redness of mucosal surface, breaks in the mucosal surface, bleeding (describe color [e.g., is it fresh blood], quantity, site/source of bleeding), ulcers (describe shape, size, location, bleeding, etc.), warts or any other lesions (look for scratches and evidence of trauma)
  Include in your inspection examination of the vaginal fornices and the cervix. For the cervix report on any discharge (describe as above), mucosal surface abnormalities such as redness, friability, ectopy, ulcers or any other lesions
  While inspecting the inside of the vagina observe and report presence of the study product (the gel), or if there is any evidence to suggest use of traditional vaginal products
- Finally perform a bimanual vaginal exam and report on any tenderness on bimanual exam, size of the uterus, presence of any masses, or other abnormal findings.

7.0 PROCEDURE FOR COLLECTION, TESTING AND STORAGE OF BLOOD SAMPLES

Purpose

To perform the following laboratory work:

- HIV serology
- Syphilis serology
- Hemoglobin measurement
- Archive plasma

When

At enrollment and subsequent visits.

By whom

Project nurses.

7.4 Supplies needed

1. Lancets
2. 5 or 7mm needle
3. 5 ml Red-top collection vacutainer
4. 10 ml Purple-top collection vacutainers
5. Hemocuvettes
6. Alcohol swabs

Description of procedures

1. For rapid HIV testing, blood is collected from a finger prick (or infant cord blood for late presenters in the NVAZ study). A study nurse will use a drop of whole blood on a rapid HIV test kit to immediately determine if a specimen is HIV positive or negative based on the manufacturer's instructions.
2. Specimens positive on a rapid HIV test are confirmed by conventional ELISA (e.g., Wellcozyme HIV test) using serum samples according to manufacturer's instructions. If a specimen is borderline, a Western blot test will be conducted to confirm ELISA results.
3. Women who are HIV tested for the first time will be tested using two separate conventional ELISA tests.
3. All HIV negative women who seroconvert during the METRO Study will be confirmed by a WB test according to manufacturer's instructions.
4. Hemoglobin is estimated on a drop of blood (collected on a hemocuvette) using a Hemocue machine. The hemoglobin results are read immediately by a project nurse.

5. Syphilis serology (RPR and TPHA) will be performed on sera collected from all participants according to manufacturers' specifications. TPHA confirmatory tests will be performed only on RPR positive samples.
6 Two aliquots of approximately 1.5 ml plasma will be archived. Only plasma will be archived - not serum.

7.6 Processing and storage

1. HIV ELISA analysis on maternal blood samples will be performed at the JHP laboratory in Blantyre. Every effort will be made to make the results of the confirmatory tests available before the woman is discharged after delivery. Delayed results will be available by Day 7 visit postnatally.
2. Archived plasma will be stored in a -70°C freezer.

8.0 PROCEDURE FOR COLLECTION AND TESTING OF VAGINAL AND URINE SAMPLES

8.1 Purpose

To collect vaginal and urine samples to test, respectively, for bacterial vaginosis (and other genital infections; see section 3.2) and pregnancy.

8.2 When

At enrollment and subsequent visits.

8.3 By whom

By the project nurses.

8.4 Supplies needed

- pH strip
- Cotton-tipped applicator swabs
- Test tube containing sterile saline
- 10% KOH
- Glass slides
- Sterile centrifuge tubes for collection of cervico-vaginal fluid (CVL)
- Pregnancy test kit

8.5 Description of procedures

1. Vaginal pH. A pH strip is held face-down against the lateral wall of the vagina with a cotton tipped applicator until moistened with vaginal fluid. The pH is read by comparing the color on the strip to the reference colors provided by the manufacturer.
2. Vaginal wet mount. A cotton-tipped applicator is used to swab the lateral wall of the vagina and placed in a test tube containing 0.5 ml of sterile saline. The fluid is examined at the lab for presence of clue cells and motile trichomonads. A Whiff test is also done on this sample. A drop of the fluid is mixed with 10% KOH for detection of yeast cells (buds and hyphae).
3. Vaginal smears. A separate cotton-tipped applicator will be used to collect vaginal and cervical swabs to prepare a smear for Gram staining. The applicators are gently rolled (not rubbed) across the surface of the a glass slide to create a single layer of cells. The slides are allowed to air dry and stored. A separate protocol will be provided for Gram-staining, examination under oil immersion, and description of bacterial vaginosis morphology.
4. Archiving of cervico-vaginal swab. A cervico-vaginal swab sample will also be archived.
5. Urine pregnancy testing. Urine is tested for the presence of hCG for pregnancy diagnosis by a rapid dipstick analysis according to manufacturers' instructions.

9. GENERAL GUIDELINES FOR SHIPMENT OF SAMPLES

When to ship
On request by Dr. Taha.

How to ship
Via DHL or other couriers, as appropriately arranged.

Procedure
To ship frozen samples, dry shippers must be primed with liquid nitrogen for at least 48 hours prior to shipping. The shipment must leave on an appropriate day (e.g., Monday or Tuesday) to ensure the shippers arrive before the end of the working week in the U.S.

Shipping list
All boxes should have a shipping list inside, which indicates each specimen's ID number. A copy of the shipping list should be filed in the Blantyre project office for future reference. Dry shippers must be labelled with the following shipping address:

Dr. Taha E Taha
Dept. Epidemiology
Johns Hopkins University
615 North Wolfe Street
Room E6140
Baltimore, MD 21205 - USA
Phone: 410-614-5255

Some important notes

- DO NOT stick the green sticker (indicating liquid nitrogen) on the shippers, since the actual shipper does not contain any liquid nitrogen anymore.

Each shipment should be accompanied by the following VALID papers:

- Department of Health & Human Services permit to import or transfer etiological agents or vectors of human disease, which MUST be in Dr. Taha's name
- A PERMIT STICKER of the Department of Health & Human Services, with permit number stuck on the dry shipper

The value of the shipment is M.K. 50.00 (customs purposes) and contents must be specified as *potentially infectious human blood specimens* (according to the shipping permit's description of material).

Dr. Taha must be informed a head of time of:

- intended date of shipment
- airway bill number
- estimated date of arrival

When shipping, please keep in mind USA public holidays; Malawi public holidays; the size of the aircraft to Harare/S. Africa (when shipping two large dry shippers at the same time; it's best to phone the Courier beforehand to enquire if these shipments can fit in the aircraft).

Always leave appropriate contact numbers with the Courier so that they can call you if a problem is encountered.

10.0 STUDY FORMS AND DOCUMENTATION PROCEDURES

10.1.1 Study Forms (See Appendix 3)

Mother's Study Forms
Enrollment Forms:
1. Locator Information
2. Enrollment History (Demographics, Behaviour, and Genital History)
3. Physical Exam
4. Product Dispensation
5. Laboratory Check-list
6. Local Laboratory Results
7. Comments

Follow-up Forms:
1. Locator Information - update only
2. Follow-up Behaviour and Genital History
3. Acceptability History (complete at test of cure visits)
4. Physical Exam
5. Product Monitoring and Genital History (complete at test of cure visits)
6. Product Dispensation
7. Laboratory Check-list
8. Local Laboratory Results
9. Missed Visit
10. Adverse Experience
11. Concomitant Medication
12. Termination
13. Comments

10.1.2 Purpose of each study form

Most of the forms are self-explanatory. Only few will be explained here. The Locator Information Form is completed at time of enrollment of the woman and updated at each follow-up visit. This form will be used to trace women in situations where participants are late or miss a scheduled visit. Therefore, complete this form with extra care, so that it serves its purpose (i.e., to locate a study participant in a particular neighbourhood or township). If you do not do it well now, you will bear the difficulties later!!! The Comment Form is an open ended form which records additional information about a specific participant, or to clarify data recorded on another form. Information about unscheduled visits is also recorded on this form. If this a scheduled visit, enter visit number on this form as usual (i.e., the current visit number). If this is an unscheduled visit, leave the visit number blank - only enter ID# and date of visit. Please write legibly on this form. The Missed Visit Form is completed when a participant has missed a scheduled visit. Enter on this form the visit # of the scheduled visit that was

missed. The Termination Form is completed whenever a participant terminates from the study. The termination date and the reason for termination (e.g., death of the mother) are entered.

10.1.3 By Whom
Study nurses.

10.1.4 Where/When
At QECH or participating clinics at the time indicated on each form.

10.1.5 Procedure
To complete these forms, follow carefully the instructions on each form. Write legibly using a black pen. Enter the responses in the boxes or lines provided. No scribbling is allowed. No wipe-outs. When a correction is made, make a neat single line crossing over the original entry; initial and date this correction and enter your code at the margin. Please follow these instructions carefully.

10.2 Clinic specific METRO study log-book

10.2.1 Purpose
To create an overall METRO study registry.

10.2.2 When
First contact of a woman (prior to enrollment) with hospital or a health center as a potential study client.

10.2.3 By whom
Study nurses.

10.2.4 Procedure
Each participating clinic will register women presenting postnatally in a designated METRO study log book. The log book will include pertinent information such as name, date and residence and any other information required at the start of the study.

10.3. Documentation procedures
The study site principal investigator will maintain all source documents used to complete the study forms (see section 10.1 above). A source document is defined as the first document on which study-related information is recorded. All data collection forms and source documents will be kept in locked files in a secure area, with access limited to study staff only.

Source documents will be kept in a locked file cabinets in the office. All locator information forms will be kept in locked files in a secure area separate from any other study document. In order to protect the participants confidentiality, under no circumstances will documents bearing participants names or other personal

identifiers be stored together with documents bearing the participants ID number.

The project will maintain a log or database containing the link between participant names and ID numbers. Written logs and/or database print outs will be stored in a secure location separate from participant files, with access limited to the Field Director and the Data Manager.

The project staff must adhere to the following standards of source documentation:

1. Although information may be copied from source documents onto other forms, or entered onto a computer database, all original source documents must be kept in the participant file.
2. The study participant whose information is contained in a source document must be identified by name or ID number, but not both, on the document.
3. All individuals who enter information onto source documents must be identified on the document, and must date all entries.
4. Change to entries on source documents must be initialled and dated by the individual making the revision. The individual's code should also be entered at the margin of the page.
5. Source documentation prepared by the project laboratory must be reviewed, signed and dated by designated technician (the tech's code number should be indicated at the margin).

11.0 STUDY TREATMENT

11.1.1 Drug supply, distribution and pharmacy

The study product and placebo are donated by 3-M Pharmaceuticals. MetroGel-Vaginal (0.75%) is an effective, well-tolerated local treatment for BV. Each gram of MetroGel-Vaginal contains 7.5 mg of metronidazole, 0.8 mg of methyparaben, 0.2 mg of propyl-paraben in an exclusive hydrogel vehicle consisting of purified water, propylene glycol, carbomer 934P, sodium hydroxide (to adjust pH), and edetate disodium. The gel is buffered to a normal pH of 4.0 and is free of mineral oil. Each applicatorful delivers about 5 grams of gel and contains approximately 37.5 mg of metronidazole in solution. The active product and placebo are identical with the exception that the placebo lacks metronidazole. The product should be stored at a temperature range of 15-30° C. An expiration date of at least two years will be ensured upon shipping the product from the U.S.

The receipt, dispensing and return of all study treatments will be documented by a designated project pharmacist, the project coordinator, on appropriate log forms.

Separate accountability records will be maintained for each shipment of drugs received by the project. A separate record will be maintained for all product supplies returned to the project unused by the participant. Assessment for compliance with product use will include return of the used product and applicators, checking the product number on returned product tube, and assessing amount of product used. These will be collected on the product dispensation and product monitoring forms that are completed at each visit.

All product inventory records will be stored in a locked file cabinet in a secure area of the project pharmacy, with access limited to the Field Director and the Project Coordinator.

11.1.2 Dosing

Each tube contains 70 g of metroGel-Vaginal. Study participants will be provided a single new product tube and a set of five new applicators at each visit. Women will use a new applicator each time the product is applied (dose 5 g per 5 ml applicator each night for five consecutive nights). The total amount of gel being used after each visit is therefore only about 25 g. Women will be instructed to bring back the tube so that the unused portion of the gel can be assessed.

Women will be instructed to use the product for 5 consecutive nights. The gel use should not be started during mensis. If mensis starts while the product is being applied, use should be discontinued and re-started when mensis stops. If a woman forgets to insert the gel for any reason, she should apply it the following night until a total of five applications have been completed (i.e., all new applicators have been used). Women should be instructed not to clean or wash

the inside of the vagina until the following morning.

11.2 Adverse experiences

11.2.1 Summary

Intravaginal metronidazole gel is recommended by the Centers for Disease Control (CDC) for treatment of BV and no major risks have been reported when used in non-pregnant women. Minor complaints include yeast infection, vaginal irritation and rah. We will perform a wet mount at each visit (and at interim visit, if necessary) to monitor these infections and will provide treatment when clinically indicated. Information on use of the gel during pregnancy is limited. Therefore, the gel will be stopped if a woman becomes pregnant while using the gel. The dose of metronidazole intravaginal gel is very small compared to a usual oral metronidazole dose (about 188 mg intravaginal compared with 5000 mg oral). Additionally, systemic absorption of intravaginal gel was reported to be very low. Because some interactions have been reported between oral metronidazole use and alcohol, we will advise women not to drink alcohol while using the gel.

11.2.2 Monitoring of treatment side effects

Women will be closely monitored for acute and long-term side effects of metronidazole gel.

11.2.3 Management of side-effects

Women will be adequately counselled about side effects and instructed to report to the clinic if further assistance is needed (unscheduled visits). The project employs a full time clinical officer to provide clinical care to study participants. Specialized obstetric and adult medical care services are also available to the study participants.

11.2.4 Documentation and reporting of adverse experience

All adverse experiences (AEs) will be documented on the Adverse Experience case report form. All serious AEs (SAE) will be immediately reported to the principal investigators (on site and in the U.S.). See Appendix 4 for assessment of Adverse Experiences.

11.2.5 Non-study drug treatment

Studies from which women are recruited into the METRO trial provide at no cost all participating women multivitamin and iron supplements to safeguard against anemia. This commences at discharge from hospital after delivery and continues postnatally. All women with reactive syphilis serology will receive appropriate treatment. The children of these women will be assessed and provided treatment if clinically indicated.

12.0 STATISTICAL CONSIDERATIONS

12.1 General design issues

This is a randomized, double masked, controlled clinical trial to determine the effect of metronidazole intravaginal gel treatment on the incidence of BV among HIV positive and HIV negative women.

12.2 Endpoints

12.2.1 Primary endpoints

- Incidence/Prevalence of BV at cross-sectional visits;
- Rate of conception and portion of pregnancies lost during the first 14 weeks of gestation;
- Incidence of adverse reactions to the product (portion of subjects having adverse reactions).

12.2.2 Secondary endpoint

- Rates of participation in study, adherence to treatment, and subsequent dropout.

12.3 Randomization procedures

HIV positive and HIV negative women are randomized to receive either the active product (intravaginal metronidazole gel) or placebo gel. Five Study Clinics, including the QECH, are participating in this trial (more clinics could be added in the future). The allocation of study IDs for each clinic are shown below. Of the 1000 IDs assigned to each clinic, 500 will be for HIV positive clients and 500 will be for HIV negative clients. Of these 500 IDs for each of the HIV positive or HIV negative clients, half will be randomized to active product and the other half to placebo. The exact number of clients each clinic will enrol will depend on sample size (see Section 12.4.1).

Clinic	Code	ID Range HIV -ve	ID Range HIV+ve
QECH	1	11001-11500	12001-12500
Limbe	2	21001-21500	22001-22500
Cholomoni	3	31001-31500	32001-32500
Zingwangwa	6	61001-61500	62100-62500
Ndirande	7	71001-71500	72001-72500

12.3.1 Instructions for using the allocation study ID's

By Whom

Study coordinator (as the rest of the study investigators and workers, the study coordinator is completely blinded about the treatment assignment).

Procedure

1. Assign these Lists to the five Study Clinics as shown in section 12.3. above.
2. Each woman enrolling in the study will be sequentially assigned the next available ID in each clinic.

12.4 Sample size and analysis plan

12.4.1 Sample size and power:

The following design specifications will apply: number of treatment groups: 2; follow-up period: approximately one year; hypotheses will be tested using a one-sided type 1 error of $\alpha=0.05$. Unless stated otherwise, powers are obtained based on Fisher's exact test for comparisons of proportions in two groups.

We will recruit 1664 women of whom 832 will be HIV infected and 832 will be uninfected. In each group of women, 416 will be randomized to treatment arm and 416 to placebo arm. Based on previous experience in Malawi, losses due to dropouts and non-compliance will be less than 10% per year. Thus at least 375 women in each arm will return to each study visit during the first year.

Primary endpoint: effect of treatment on BV incidence. Power estimates for this primary endpoint are shown in Tables 12.1. In previous studies from Malawi, cross sectional prevalence of BV among untreated women was 30% and that of any vaginal disturbance was 89%.

Table 12.1. Estimated power for specific aim 1 (comparing BV at each visit during the first study year assuming at least 375 evaluable study subjects in each group).

**Normative rate in untreated women**	**Reduction from intervention**	
	**33%**	**50%**
0.30	**.87**	~1.00
0.89	**~1.00**	~1.00

Thus there will be **0.87** and almost **1.00** power to detect 33% reductions in cross sectional visit prevalence of BV (normative rate 0.30 in untreated subjects) and any vaginal flora disturbance (normative rate .89 in untreated subjects).

Primary endpoint: effect of treatment on rates of conception and early pregnancy loss. Previous reports suggest that 20%-30% of women without treatment will lose their first post-index pregnancy. Table 12.2. presents powers to detect 33% and 50% reductions in the rates of early gestational loss of this first post index pregnancy based on 300 evaluable subjects in each group (we have assumed that 80% of these women will become pregnant again). There is virtual certainty to detect 50% reductions if pregnancy loss of 30% occurs, and >90% reduction if the rate of pregnancy loss is 20%. For a 33% reduction, there is about 80% power if the rate of loss is high (30%) and limited power if the rate of pregnancy loss is low (20%).

Table 12.2. Estimated power for specific aim 2 assuming 300 evaluable subjects in each group during the first year.

Normative rate of pregnancy loss	Reduction from intervention 33%	50%

12.5 Data management

Data entry and storage will be conducted on-site. All completed forms (source documents) are checked for completeness before the participant leaves the ward or clinic by a nurse other than the nurse who initially completed the forms. After ensuring no questions are left blank, the forms are accumulated and sent to the data entry office at the end of each day. Data clerks using FoxPro programs will double enter all forms. The Data Manager will run verification programs and SAS frequencies to check for errors and out of range entries. After all data entry has been completed, the forms are archived in numeric order in locked cabinets in the data entry office. In addition to local data management and analysis, the data will electronically be transferred to Baltimore for further management and analysis.

12.6 Data analysis.

Analyses will be done using an intention to treat approach. Separate analyses for HIV infected and uninfected women will be performed and the effect of the intervention on outcomes in these two groups will be compared. The effect of the intervention will be based on comparing incidence [1] of BV and portion of pregnancy losses between treated and untreated (control) subjects.

Multiple hypotheses will be tested in this study, requiring a diverse array of analytical methods to compare the treatment and control arms. Descriptive comparisons will be made between the two study groups to identify potential confounding variables which could bias hypothesis testing through direct comparisons. When possible, as differences are noted, multivariate adjustment methods will be used to minimize the effect of confounders on testing the hypotheses. Continuous variables will be tested for skewness, kurtosis and outliers which could cause imprecision in standard asymptotic statistical methods. When such conditions occur, transformations such as logarithms, dichotomization or Winsorizing will be used, or nonparametric statistical methods will be implemented. For the statistical tests described below, both P-values and confidence intervals will be calculated for the parameters of interest.

[1] *Although we use the term incidence in the text, it is more accurate to define this measure as cross-sectional prevalence because the number of BV cases during a 3 month interval are a combination of new and continuing cases (due to incomplete cure and recurrences).*

Most outcomes of interest (e.g., presence of BV) are uncensored binary or categorical events. These include demographic and specific behavioral factors, etc. Contingency (n x 2) tables will be used to compare these events between the two study arms. Adjustment for covariates will be made with logistic regression. Comparison of binary events between two time-points within the same group will be done using methods that match on the same individual such as discordant pairs or conditional logistic regression. In other outcomes of interest which are continuous comparison of mean values will be made using t-tests. Adjustments for covariates will be performed through linear regressions. Comparisons of continuous covariates for the same individuals in a group over different time periods will be made using paired z-tests and linear regressions on differences of values.

Some outcomes will be censored binary events. Standard survival analysis techniques such as Kaplan-Meier curves and Poisson regression will be used to directly compare outcome in both groups. These methods can also compare incidence rates within the same group over different time periods. Multivariate analogues that utilize covariates (proportional hazards models and Poisson regression) will adjust these comparisons for other covariates.

For some analyses we will pool repeated measurements from the same individual over multiple study visits. For example, occurrence of BV across all post-intervention study visits will be compared between study groups. For these analyses, generalized estimation equation models, which use an individual as cluster unit, will be fit to obtain robust covariance. For binary outcomes, a logit link will be used.

12.7 <u>Data monitoring</u>

This trial is monitored by a local Data and Safety Monitoring Board (DSMB). The members of the DSMB have no vested interest in this study. An initial administrative/safety analysis will be performed after the first three months of accrual. This will be followed by formal analyses of safety and efficacy of the intervention, based on the primary endpoints. Rates of accrual, retention, adherence and adverse events will be closely monitored and presented to the DSMB.

12.8 <u>Blinding rules</u>

This is a double-masked clinical trial. Blinding of study participants will remain in place until the study is completed (after all data have been entered into the study database and all queries have been settled). However, unblinding can be considered if the clinician providing service decides that it is necessary to know the exact treatment the client has been receiving. Under such circumstances, the Field Director will contact the PI to arrange with the study statistician to make this information available. Participants will be informed of their treatment assignment only at the study completion. To preserve the integrity of the study, participants who are terminated early (e.g., for having completed the study visits schedule) will wait until every woman has been enrolled and completed the study.

13.0 ADDENDUMS

13.1 Study population

Women who have been screened for HIV in other studies such as the HIVNET 016A condom promotion and Mastitis studies are eligible for enrollment. Likewise, women of reproductive age who are sexually active (e.g., women attending family planning clinics, postnatal and under-five clinics) are also eligible as long as they agree to be HIV tested. All prospective participants should not be pregnant. This modification opens enrollment to all women who satisfy inclusion criteria. The study is not restricted to postnatal women.

13.2 Screening consent form

A new consent form has been developed and added to this study forms to obtain consent for HIV testing. This is intended only for women who were not tested previously. This HIV screening process will precede enrollment. The enrollment consent form will be separately completed on all women.

13.2 Inclusion/Exclusion criteria

Women who are sexually active and 18 years or older will be eligible.

13.3 Women who become pregnant during the trial

First, product use will be stopped. Second, these women will be followed throughout their pregnancy and will attend the study visits as scheduled. These women will be followed to three months after delivery. Events during pregnancy and at delivery (including infant birth weight, mode of delivery, immediate postantal events such as sepsis) will be documented.

13.4 Pharmacy

- Product will be ordered directly from the pharmacist designee (Mr Gondwe).
- Order one tube at a time
- For follow-up visits, order the visit-specific tube 2 weeks ahead of the scheduled visit
- Three forms to be completed to document ordering, dispensation and inventory
- At enrollment the nurse will use an extra applicator to demonstrate to the woman how to fill the applicator with the gel and how to apply the applicator into the vaginal. The amount of the gel in the tube is adequate to perform this demonstration.
